# The Effects of Ocean Warming and Acidification on Fatty Acid Contents of Marine Organisms: A Global Meta‐Analysis

**DOI:** 10.1111/gcb.71056

**Published:** 2026-08-19

**Authors:** Bethany Rose Dawson, Evangeline Mantzioris, Ivan Nagelkerken, Sean D. Connell, Camille Mellin

**Affiliations:** ^1^ School of Biological Sciences Adelaide University Adelaide South Australia Australia; ^2^ School of Allied Health and Human Performance Adelaide University Adelaide South Australia Australia; ^3^ South Australian Research and Development Institute (Aquatic Sciences) Henley Beach South Australia Australia

**Keywords:** climate change, DHA, EPA, micronutrients, nutrition security, omega‐3

## Abstract

Ocean warming and acidification pose significant threats to marine biodiversity and human nutrition by fundamentally altering the biochemical composition of marine organisms. A primary concern is the potential decline in omega‐3 fatty acids (FAs) that are essential to human health and primarily obtained through seafood consumption. The influence of these climate stressors on FA content across marine food webs remains poorly understood. To address this critical knowledge gap, we conducted a global meta‐analysis of 489 experiments across 143 publications and 132 marine species, quantifying the effects of warming and acidification on nutritionally important FAs in marine primary producers, invertebrates and fishes. Under warmer conditions, we detected reductions of up to 34% and 50% in omega‐3 FA proportions and concentrations, respectively, and up to 53% in omega‐3: omega‐6 ratios, together with increases of up to 22% and 17% in saturated FA proportions and concentrations, respectively across taxa groups. Critically, these effects were intensified as warming increased. The most severe reductions in omega‐3 FAs were observed in primary producers, suggesting that climate‐driven changes at the base of the food web could impair trophic transfer of these micronutrients, limiting nutrient availability for higher trophic levels and ultimately humans. Ocean acidification, conversely, demonstrated a minor overall effect on FA levels, although this result was based on substantially fewer studies. Furthermore, we identified species from environments with broader temperature ranges, diatoms, herbivorous invertebrates and low‐resilience fish to display larger reductions in omega‐3 FA proportions under warming. Our meta‐analysis further highlighted the need for long‐term studies under ecologically realistic conditions to improve predictions of nutritional responses to climate change. These findings are essential for understanding how the nutritional value of species change under climate change, and can inform fisheries management, aquaculture and public health policy aimed at securing the future availability of these vital micronutrients.

## Graphical abstract

This global meta‐analysis across 132 marine species found that ocean warming decreases nutritional quality by reducing polyunsaturated fatty acids, including the health‐critical omega‐3 fatty acids EPA and DHA. These changes lead to declines in omega‐3: omega‐6 ratios of up to 53%, and increases in saturated FAs of up to 21%, with shifts strongest in primary producers. Responses were partly explained by biological and methodological factors, including taxon, exposure duration, climate zone and life stages, whereas acidification effects were generally weaker and less consistent. These shifts have widespread implications for trophic transfer and human nutrition globally throughout marine food webs.

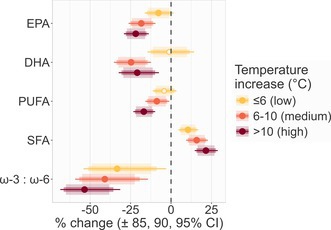

## 1 Introduction

The world's oceans are vital for food web functioning and human health, playing an indispensable role in global food security by supplying billions of people with essential nutrients, particularly long‐chain fatty acids (FAs) (FAO 2024; Golden et al. 2016). However, the continued ability of oceans to provide these nutritional benefits is increasingly threatened by multiple stressors, with climate change encompassing some of the most pervasive and complex threats. Ocean warming and acidification are now established global trends; since 1980, ocean temperatures have risen by 0.6°C and pH has decreased by up to 0.027 units per decade, and the frequency of marine heatwaves has doubled (IPCC 2023). By 2100, average sea surface temperatures are projected to rise up to 4.1°C since pre‐industrial conditions, with pH declining by 0.44 units under a business‐as‐usual emission scenario (IPCC 2023). Marine heatwaves are also expected to become up to 8 times more frequent and increase in both duration and intensity (IPCC 2023). These profound environmental shifts are already altering species distributions, community composition and functioning (Nagelkerken and Connell 2015; Poloczanska et al. 2013; Smale et al. 2019), with significant, yet poorly characterised consequences for the biochemical compositions of marine organisms (Hixson and Arts 2016).

Marine organisms provide the exclusive natural source of the crucial long‐chain omega‐3 polyunsaturated FAs (PUFAs): eicosapentaenoic acid (EPA) and docosahexaenoic acid (DHA). These FAs are foundational for essential biological processes in marine organisms and terrestrial vertebrates, and are critical for human health, supporting everything from growth, reproduction and metabolism to anti‐inflammatory immune functions (Calder 2018; Lund 2025; Pilecky et al. 2021). DHA is particularly important for early development and brain and eye function, whilst both EPA and DHA are associated with reduced risk of cardiovascular and other chronic diseases (Calder 2018; Fisheries Research and Development Corporation (Australia) 2004).

Marine primary producers (e.g., micro‐ and macroalgae) are uniquely capable of de novo synthesis of EPA and DHA, allowing these vital FAs to accumulate through trophic transfer across the entire marine food web (Gladyshev et al. 2013; Hixson et al. 2015). Conversely, terrestrial plants only produce short‐chain FA precursors (e.g., α‐linolenic acid, ALA) (Gladyshev et al. 2013; Hixson et al. 2015). Most marine fish have lost enzymatic capacity to convert ALA to EPA and DHA, possibly due to abundant dietary availability of long‐chain PUFA in the marine environment, whilst biosynthesis capabilities in marine invertebrates appear highly variable yet are poorly understood (Monroig et al. 2022; Pereira et al. 2003). In humans, conversion of ALA is generally inefficient, making dietary intake of preformed EPA and DHA advantageous (Hixson et al. 2015; Pilecky et al. 2021).

Temperature and pH shifts can alter cellular membrane properties, which may prompt physiological responses that affect FA composition (Hixson and Arts 2016; Rossoll et al. 2012). Under increased temperatures, organisms increase cell membrane saturated FA (SFA) content to maintain structural rigidity, whilst reducing the content of more fluid unsaturated FAs like EPA and DHA; a process termed homeoviscous adaptation (Sinensky 1974; Farkas 1979). Additionally, warming reduces dissolved oxygen, potentially triggering changes in enzyme activity and metabolic pathways that favour omega‐6 FA synthesis at the cost of omega‐3 FAs, reducing the omega‐3: omega‐6 ratio (Calder 2010; Hixson and Arts 2016). Acidification can also affect enzymes involved in FA synthesis and desaturation (the conversion of SFAs to unsaturated FAs), with lower pH potentially promoting SFA accumulation to reduce membrane permeability to CO_2_, thus stabilising intracellular pH (Rossoll et al. 2012).

These biochemical changes are not only ecologically significant but raise wider concerns for food nutritional quality and public health (Araujo et al. 2021). Over half of the global population may consume suboptimal levels of EPA and DHA (Colombo et al. 2020; Micha et al. 2014; Murphy et al. 2015). This issue is compounded by an imbalance in dietary omega‐3 and omega‐6 intake, particularly in Western diets that are typically high in land‐based foods rich in omega‐6 (Ciesielski 2025; Simopoulos 2002). Omega‐3 and omega‐6 pathways share enzymes, meaning excessive omega‐6 intake can competitively inhibit omega‐3 synthesis and utilisation (Calder 2015; Simopoulos 2002). Consequently, declining EPA and DHA in marine species may cascade through food webs, reducing the nutritional quality of seafood (Feijão et al. 2018; Hixson and Arts 2016).

Although many studies have investigated how temperature affects FA levels in marine organisms, findings are often highly variable and context dependent, making it challenging to identify consistent patterns and draw broader implications. Responses to warming likely vary with biological traits that influence metabolic demands and physiological adaptability such as habitat type, climate zone and life stage (Antonucci et al. 2019; Garzke et al. 2017; Lloret 2010). However, the extent to which these factors moderate FA responses remains poorly understood, restricting our ability to project future availability of long‐chain FAs and predict which taxa or regions are most vulnerable to nutritional shifts. Comparisons across studies are further limited by FA inconsistencies in FA reporting, with proportions and concentrations of various units used alternatively. FA proportions help assess nutritional quality and omega‐3 to omega‐6 balance; however, without concentration data that reflect absolute FA amounts, conclusions about overall nutrient availability remain incomplete. Both metrics are important for fully understanding how ocean change alters FA dynamics.

Our meta‐analysis quantifies how ocean warming and acidification affect FA levels in marine organisms, with a focus on omega‐3 FAs critical to ecosystem and human health. Specifically, we (i) assess the direction and magnitude of warming and acidification‐induced changes in FA levels across marine primary producers, invertebrates and fish under controlled experimental conditions and (ii) identify biological and methodological factors that modulate these effects. We examine changes of key FAs using both proportions (i.e., percentage of total FAs), and absolute concentrations to provide complementary insights. By analysing both metrics, this study provides a comprehensive understanding of how ocean warming and acidification may influence the nutritional quality of marine organisms.

## 2 Materials and Methods

A systematic literature search was carried out following stringent criteria (Figure S1). When these criteria were met, FA data were extracted along with biological trait data and methodological information. The effects of warming and acidification were calculated within studies using the log response ratio of the means (ln*RR*) and multilevel meta‐analytic mixed effect models were fitted to investigate both aims. Firstly, models assessed the overall effect of warming or acidification on FA levels among three taxa groups: primary producers, invertebrates and fish. Secondly, models examined how the effect of warming on FA levels varied across biological and methodological factors for each taxa group. All data manipulation, statistical analyses and plotting were conducted in the R programme (version 4.4.2) (R Core Team 2024). ChatGPT (May 2024, version 4o) by OpenAI was used to improve the readability of the manuscript.

### 2.1 Study Selection

Relevant scientific peer‐reviewed studies with data on the effect of warming or acidification on FAs of marine organisms were identified using the search engines ‘Scopus’ and ‘Web of Science Core Collection’ on 13th April 2022. The search terms used were: (‘Marine’ OR ‘Sea’ OR ‘Seafood’ OR ‘Ocean*’ OR ‘Estuar*’) AND (‘Climate change’ OR ‘Warming’ OR ‘Acidification’ OR ‘Temperature*’) AND (‘Fatty acid*’ OR ‘Omega‐3*’ OR ‘PUFA’). Searches were conducted across study titles, abstracts and keywords. To be included in the meta‐analysis, studies needed to measure the FA concentrations and/or proportions of a given eukaryotic taxon held alive in at least two controlled marine environments of different temperatures or acidification levels during the postembryonic stage. Studies had to report at least one of the following: ALA (18:3n‐3), EPA (20:5n‐3), DHA (22:6n‐3), total omega‐3, Linoleic acid (LA; 18:2n‐6), AA (20:4n‐6), total omega‐6, total PUFA, total monounsaturated FA (MUFA), total SFA, total FA or the omega‐3: omega‐6 ratio. Studies were excluded based on a set of strict criteria (Figure S1), applied first during title and abstract screening and subsequently during full‐text screening. If the sample size was one or unspecified, data were not included unless the FA analysis was performed on a pooled sample (e.g., combined individuals or colonies). In such cases, the data were included but assigned a sample size of one. When there was uncertainty in whether the criteria were met, authors were contacted via email for clarification and requests for unpublished datasets were made where not publicly available. Of the 30 authors contacted, 10 responded and data from five were subsequently included in the meta‐analysis.

### 2.2 Data Extraction

FA amounts and units were recorded from text, tables and figures, along with corresponding sample sizes, standard deviations (SDs) or standard errors (SEs). When data were only presented graphically, values were extracted using WebPlotDigitizer (Rohatgi 2022). When the sample size was reported as a range, the mid‐point was used for weighting and variance calculations. When the omega classification (e.g., omega‐3 or omega‐6) was not reported, assumptions were made to help assign omega groups to FAs (Supplementary Methods).

Where possible, missing SD were calculated from available data, and concentrations, proportions, ratios and totals were calculated from one another when sufficient data were provided. Measures of variance (i.e., SD) and sample sizes are both needed to conduct a weighted meta‐analysis, thus any values still missing were imputed to minimise data exclusion, following recommendations from previous studies (Nakagawa, Noble, et al. 2023; Kambach et al. 2020; Idris and Robertson 2009). For full procedures, see Supplementary Methods.

Biological traits and methodological information were collected for use as moderators in the meta‐analytic models. Species and higher order names were validated using the World Register of Marine Organisms (WoRMs) (Ahyong et al. 2025) to ensure accurate classification. Taxa were sorted into groups of either primary producers, invertebrates or fish. Biological trait data were then collected for each taxon preferentially from the primary study, but if not available, AlgaeBase (Guiry and Guiry 2025), SealifeBase (Palomares and Pauly 2025), FishBase (Froese and Pauly 2023) and WoRMs (Ahyong et al. 2025) were consulted, followed by peer‐reviewed studies if data were still missing. Traits included body size class (Figure S2a), climate zone, diet group, feeding type, functional group, habitat, longevity, mean preferred temperature, preferred temperature range, resilience to fishing pressure (Musick 1999), trophic level, vulnerability to fishing (Cheung et al. 2005) and vulnerability to climate change (Jones and Cheung 2018) (Supplementary Methods). The life stage, or growth phase in the case of many microalgae studies, at the time of sampling was also documented (Supplementary Methods). Biological traits were only included as moderators for a specific taxon group when sufficiently available and informative (Table S1). All biological trait data were collected at the species‐level, except for habitat type and body size class, which were inferred from genus‐level information using the mode when species‐level data were unavailable. Therefore, taxa not identified to species or genus level were excluded from models requiring such data. Data on maximum depth were initially collected but later excluded due to lack of availability across taxa.

Methodological data included the temperature or acidification (Supplementary Methods) of each treatment and the duration of exposure; when duration was reported as a range, the midpoint was taken. The tissue type was recorded, with whole organisms assumed when unspecified. Any additional covariates investigated alongside warming or acidification were also noted.

### 2.3 Data Manipulation

FA data from the same paper and taxon were grouped into a single ‘experiment’ (*n* = 489) when exposure duration differed by < 3 months, and all biological trait moderators, tissue type and other controlled experimental covariates were constant. FA data were only retained when levels were > 0. For each experiment, the lowest warming or acidification group was designated as the control, whilst higher warming or acidification groups served as comparison treatments. To ensure a single control per experiment, only control data with the longest duration were retained. Finally, for each control and treatment comparison, the exposure duration was calculated as the average of the two corresponding durations. This value was then assigned to one of three subcategories, defined separately for primary producers, invertebrates and fish, based on the data distribution to ensure approximately equal sample sizes (Figure S2b).

### 2.4 Calculating Effect Sizes

Effect sizes for each comparison row (control vs. treatment) were computed using the ‘escalc’ function in the ‘metafor’ R package (Viechtbauer 2010). The log response ratio of the means (ln*RR*) was calculated because it is multiplicative and does not assume homogeneity of variance between treatment and control groups, a condition not met in this dataset (Spake et al. 2023). The effect size variance (*Var*) was calculated alongside ln*RR* for model weighting and a correction factor for small sample size was applied to both metrics (Lajeunesse 2015; Senior et al. 2020) (Equations 1 and 2). No evidence of publication bias was detected, and sensitivity analyses found results to be robust to the influence of individual studies (Figure S3; Table S2; Supplementary Methods).

(1) $$\ln\mathrm{RR}=\ln\;\left(\frac{{\text{mean}}_{T}}{{\text{mean}}_{C}}\right)+\frac{1}{2}\;\left(\frac{{\mathrm{SD}}_{T}^{2}}{N_{T}{\text{mean}}_{T}^{2}}-\frac{{\mathrm{SD}}_{C}^{2}}{N_{C}{\text{mean}}_{C}^{2}}\right)$$

(2) $$\mathrm{Var}=\frac{{\mathrm{SD}}_{T}^{2}}{N{\text{mean}}_{T}^{2}}+\frac{{\mathrm{SD}}_{C}^{2}}{N_{C}{\text{mean}}_{C}^{2}}+\frac{1}{2}\;\left(\frac{{\mathrm{SD}}_{T}^{4}}{N_{T}^{2}{\text{mean}}_{T}^{4}}+\frac{{\mathrm{SD}}_{C}^{4}}{N_{T}^{2}{\text{mean}}_{C}^{4}}\right)$$

where *C* = control group; *T* = treatment group; and *N* = sample size.

### 2.5 Statistical Analyses

#### 2.5.1 Mixed‐Effect Models

Multilevel meta‐analytic models were fitted to determine (i) the overall effect of warming or acidification on FA levels within three taxa groups and (ii) which moderators best predict the effect of warming on FA levels. Specifically, we used mixed‐effect restricted maximum likelihood models implemented using the ‘rma.mv’ function in the ‘metafor’ package (Viechtbauer 2010). Separate models were fitted for each taxa group and FA combination, with effect sizes weighted using the inverse of the heterogeneity of the true effects ($\tau^{2}$) plus Var (Viechtbauer 2010) (Equation 3).

(3) $${\text{Weight}}_{i}=\frac{1}{\tau^{2}+{\mathrm{Var}}_{i}}$$

Models used a *t*‐distribution to test model coefficients and confidence intervals to avoid inflation of Type I errors when meta‐analysing a small number of studies (Nakagawa, Yang, et al. 2023). To account for non‐independence of effect sizes among studies, taxa and control groups, these terms were included as random effects in all models. To capture within‐study variation, the identity of unique effect sizes was also fitted as a random effect (Nakagawa, Yang, et al. 2023). Model goodness‐of‐fit was assessed using conditional *R* ^2^ (*R* ^2^_*c*_) values, representing the proportion of variance explained by both fixed and random effects, and residual heterogeneity was also assessed (Nakagawa and Santos 2012) (Supplementary Methods).

Reported effects were estimated marginal means (±SE) unless otherwise stated, calculated using the ‘emmeans’ R package (Lenth 2023). These model‐derived means are adjusted for other model terms, where applicable, and converted to percentage changes in FA levels for improved interpretability (Equation 4).

(4) $$\%\text{change}=100\times \left(e^{\ln\mathrm{RR}}-1\right)$$

#### 2.5.2 Effect of Warming and Acidification on FA Responses

The overall effect of warming and acidification on FA proportions and concentrations for primary producers, invertebrates and fish was estimated using separate mixed‐effect models for each taxa group, where ln*RR* was modelled as a function of the magnitude of warming or acidification (Equation 5). For each model, the distribution of ln*RR* was approximately normal; thus, no transformations were applied (Figure S4). The magnitude of warming or acidification was grouped into three categories, defined based on the distribution of all data across taxa (Figure S5). The warming levels were: ≤ 6.5°C (low), 6.5°C–10.5°C (medium) and > 10.5°C (high), whilst acidification levels were: ≤ 522 μatm (low), 522–1100 μatm (medium) and > 1100 μatm (high). Support for warming and acidification induced changes was assessed based on whether 85%, 90% or 95% confidence intervals excluded zero. Results were considered to have very strong, strong and moderate support at the 95%, 90% and 85% confidence levels, respectively.

(5) $$\ln\mathrm{RR}\sim \text{warming}\;\left(\text{or acidification}\right)\;\text{level}+\left(1\;\right|\;\text{paper}\;\mathrm{ID}/\text{taxon}\;\mathrm{ID}/\text{control}\;\mathrm{ID})+\left(1\;\right|\;e\text{ffect size}\;\mathrm{ID})$$

#### 2.5.3 Influence of Moderators on FA Responses to Warming

To assess whether specific biological or methodological moderators contribute to heterogeneity in FA responses to warming, mixed‐effect models including each moderator were fitted separately for each taxa group. Acidification effects were not included in these analyses due to insufficient data across subcategories. Ln*RR* was modelled as a function of warming level and an additional single moderator (Equation 6). Each moderator was tested individually to enable direct comparisons across taxa, given inconsistencies in available and meaningful moderators among groups (Table S1).

(6) $$\ln\mathrm{RR}\sim \text{warming}+{\text{covariate}}_{i}+\left(1\;\right|\;\text{paper}\;\mathrm{ID}/\text{taxon}\;\mathrm{ID}/\text{control}\;\mathrm{ID})+\left(1\;\right|\;\text{effect size}\;\mathrm{ID})$$

Continuous moderators were standardised to a mean of 0 and SD of 1, and significance of the slope (*β*) was assessed (*p* < 0.05). For discrete moderators, ANOVAs were implemented and if significant (*p* < 0.05), post hoc pairwise comparisons of estimated marginal means were conducted to assess differences among subcategories using the ‘emmeans’ R package (Lenth 2023), adjusted using Tukey's method. For reporting, slopes (*β*) were used for continuous moderators, whilst estimated marginal means, averaged across warming levels, were used for discrete moderators to provide model‐adjusted predictions for each subcategory.

## 3 Results

### 3.1 Data Representation

The final data set consisted of 143 primary publications (Warming [OW]: 124; Acidification [OA]: 35) with a total of 489 experiments (OW: 401; OA: 88) and 132 identified species (OW: 113; OA: 38) (Table S3). FA proportions were available in 94% of papers (OW: 94%; OA: 94%), whereas concentrations were reported in 55% of papers (OW: 52%; OA: 57%). Of the identified species, 80 (OW: 71; OA: 20) were primary producers, 33 (OW: 26; OA: 13) were invertebrates and 19 (OW: 16; OA: 5) were fish. An additional 32 primary producers (OW: 29; OA: 3) and four invertebrates (OW: 3; OA: 1) were identified only to genus level and one primary producer identified only to phylum level (OW: 1; OA: 0). Each FA group was represented by 7–451 effect sizes in primary producers (OW: 40–451; OA: 7–68), 4–89 in invertebrates (OW: 16–89; OA: 4–59) and 6–105 in fish (OW: 16–105; OA: 6–9) (Tables S4–S6). Due to limited data, models were not fitted to assess acidification effects on ALA, total omega‐3 and total omega‐6 proportions and concentrations, LA concentrations or the omega‐3: omega‐6 ratio in fish.

### 3.2 Effect of Warming on FA Proportions and Concentrations

**FIGURE 1:**
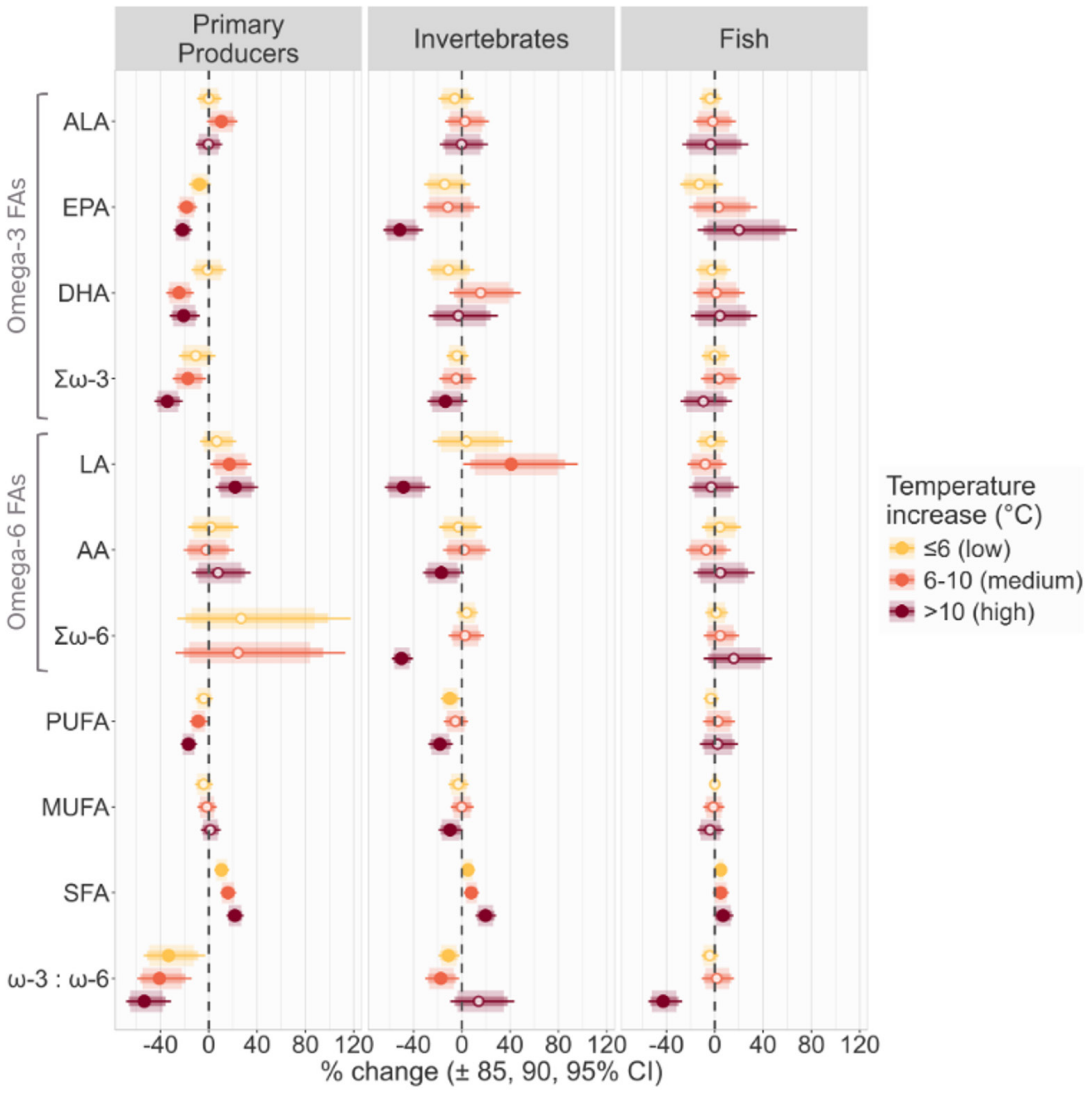
Percentage changes in fatty acid (FA) proportions (% of total FAs) in response to three warming levels (low: ≤ 6°C; medium: 6°C–10°C; high: > 10°C) calculated from average log response ratio (ln*RR*) slope coefficient estimates (*β*). The thick, medium and narrow horizontal bars denote 85%, 90% and 95% confidence intervals, respectively, and the dashed vertical line represents a null effect. Estimates to the right of zero indicate increases under warming, whilst those to the left indicate decreases. Results were considered to have very strong, strong and moderate support at the 95%, 90% and 85% confidence levels, respectively. Open circles show effects whose 85% confidence intervals overlap zero, indicating no consistent directional effect. Species represented across all models: Primary producers (*n* = 67), invertebrates (*n* = 26), fish (*n* = 14). With ALA: α‐linolenic acid (C18:3n3); EPA: Eicosapentaenoic acid (C20:5n3); DHA: Docosahexaenoic acid (C22:6n3); LA: Linoleic acid (18:2n3); AA: Arachidonic acid (20:4n6); PUFA: Polyunsaturated FAs; MUFA: Monounsaturated FAs; SFA: Saturated FAs.

Primary producers exhibited strong changes in FA proportions, with generally larger effect sizes at higher warming levels (Figure 1). There was very strong support for SFA increasing by 10%–22% across all warming levels and total PUFA decreasing by 9%–17% under medium (6°C–10°C) and high (> 10°C) warming. Omega‐3 FA also declined, with total omega‐3, EPA and DHA declining by 8%–34% across warming levels with strong to very strong support. In contrast, ALA increased by 10% under medium warming with strong support. Omega‐6 FAs generally increased, with very strong support for LA increases of 17%–22% at medium and high warming. Omega‐3: omega‐6 ratios declined by 34%–53% across all warming levels with very strong support (Figure 1).

In invertebrates, similar trends were observed, with effects generally more pronounced and better supported under high warming than low (≤ 6°C) or medium warming (Figure 1). SFA proportions increased by 5%–20% across all warming levels with strong to very strong support, whilst PUFA declined by 10%–18% at low and high warming with very strong support. There was moderate support for a 14% decline in total omega‐3 and very strong support for a 51% decline in EPA under high warming. In contrast to primary producers, omega‐6 proportions declined under high warming in invertebrates, with LA, AA and total omega‐6 decreasing by 17%–50% with strong to very strong support. The omega‐3: omega‐6 ratio decreased by 11%–17% under low and medium warming with very strong support (Figure 1).

In fish, SFA proportions increased by 5%–7% across all warming levels with moderate to very strong support (Figure 1). No significant changes were observed in PUFA, omega‐3 FAs or omega‐6 FAs. However, the omega‐3: omega‐6 ratio declined by 43% under high warming with very strong support (Figure 1).

FA absolute concentrations generally responded to warming similarly to proportions, with some notable exceptions (Figure S6; Supplementary Results). In primary producers, responses tended to show weaker statistical support than in invertebrates and fish, although trends mostly mirrored those observed across FA proportions. In invertebrates, total FA concentrations declined by 26%, total omega‐3 by 36% and EPA by 49% with moderate to very strong support under high warming, and total omega‐6 increased by 15% under low warming. In fish, FA concentrations declined by up to 54% across FA groups, including very strong support for a 50% decline in DHA and 43% decline in total FAs under high warming (Figure S6, Supplementary Results).

All models (*n* = 66) across proportion and concentration data showed significant residual heterogeneity, which was high (*I* ^2^_total_ > 75%) in 64/66 models, highlighting the importance that different study designs had on FA responses (Table S7). The sources of residual heterogeneity varied widely across studies, taxa, experiments and effect sizes, and 55/66 models explained ≥ 50% of variation in ln*RR* (*R* ^2^_*c*_) (Table S7; Supplementary Results).

### 3.3 Effect of Acidification on FA Proportions and Concentrations

**FIGURE 2:**
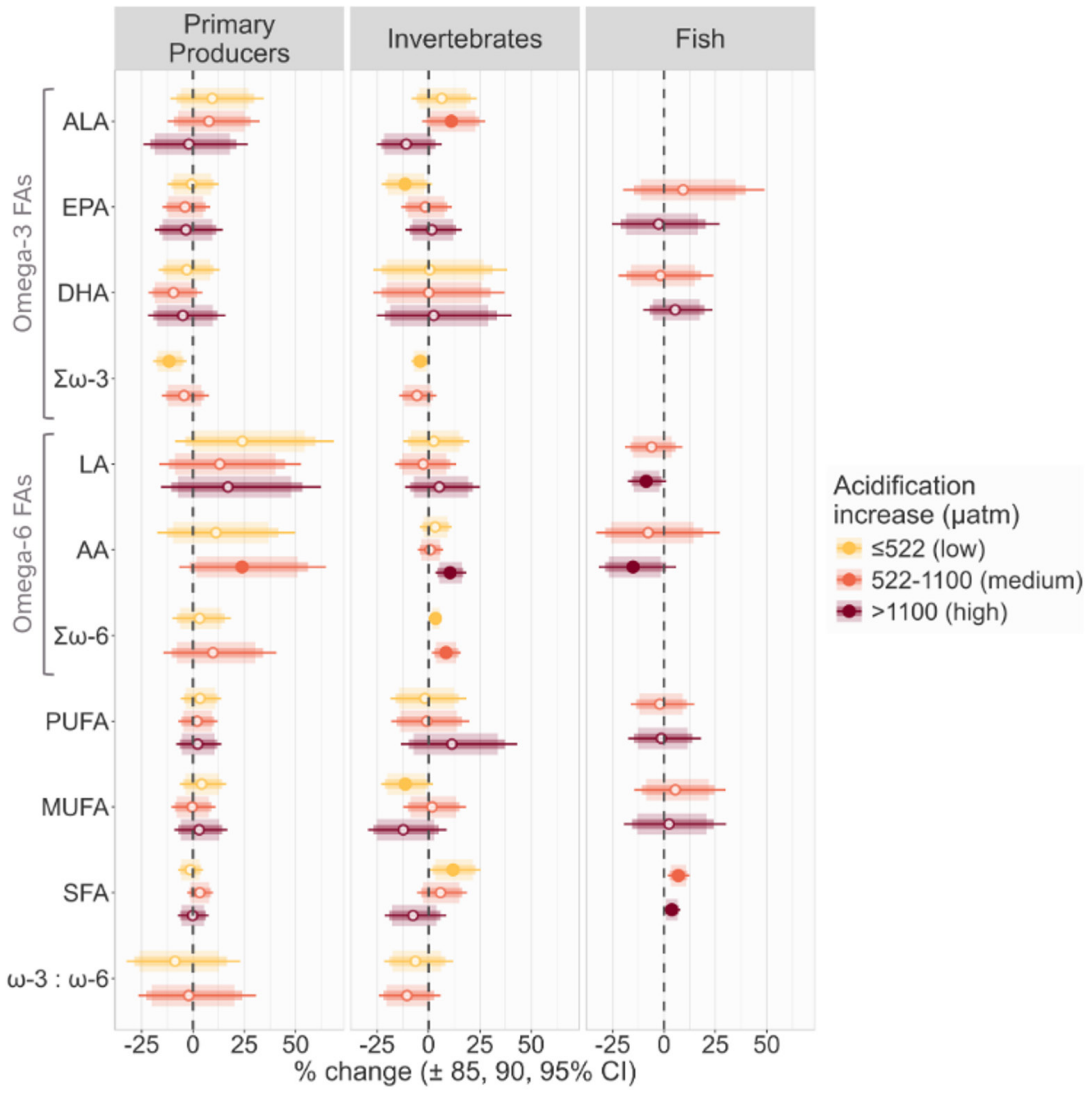
Percentage changes in FA proportions (% of total FA) in response to three acidification levels (low: ≤ 522 μatm; medium: 522–1100 μatm; high: > 1100 μatm) calculated from average log response ratio (ln*RR*) slope coefficient estimates (*β*). The thick, medium and narrow horizontal bars denote 85%, 90% and 95% confidence intervals, respectively, and the dashed vertical line represents a null effect. Estimates to the right of zero indicate increases under warming, whilst those to the left indicate decreases. Results were considered to have very strong, strong and moderate support at the 95%, 90% and 85% confidence levels, respectively. Open circles show effects whose 85% confidence intervals overlap zero, indicating no consistent directional effect. Species represented across all models: Primary producers (*n* = 19), invertebrates (*n* = 12), fish (*n* = 5). FA acronyms as per Figure 1.

In primary producers, PUFA, MUFA, SFA and individual omega‐3 (ALA, EPA, DHA) proportions showed no significant changes in response to acidification (Figure 2). However, total omega‐3 decreased by 12% under low (≤ 522 μatm) acidification with very strong support, and AA increased by 24% at medium (522–1100 μatm) acidification with moderate support. In invertebrates, SFA increased by 12% at low acidification with very strong support. No changes to omega‐3 groups were well supported except for the mixed responses in ALA which declined by 11% under high (> 1100) acidification with marginal support (85% confidence interval slightly overlaps zero) and increased by 11% under medium acidification with moderate support. Total omega‐6 increased by 3%–9% under low and medium acidification, and AA increased by 11% under high acidification with very strong support. For fish, SFA proportions increased by 4%–7% under medium and high acidification, with strong to very strong support. No changes to any omega‐3 groups were observed in fish, but LA and AA omega‐6 proportions decreased by 9%–15% under high acidification with moderate to strong support (Figure 2).

In primary producers, FA concentration data revealed declines in total FA of 15% and DHA of 24% under high acidification with moderate support and more consistent increases of 10%–36% in MUFA, SFA, EPA, LA and AA under low to medium acidification (Figure S7; Supplementary Results). In invertebrates, PUFA concentrations increased by 69% and EPA by 34% under high acidification with very strong support. In fish, concentration data were limited but indicated moderate support for declines of 19%–22% in PUFA and DHA under medium acidification (Figure S7).

Residual heterogeneity was significant in 46/58 models across proportion and concentration data, and ≥ 50% of variance in ln*RR* (*R* ^2^_*c*_) was explained in 46/58 models (Table S8; Supplementary Results).

### 3.4 Influence of Moderators on FA Responses to Warming

**FIGURE 3:**
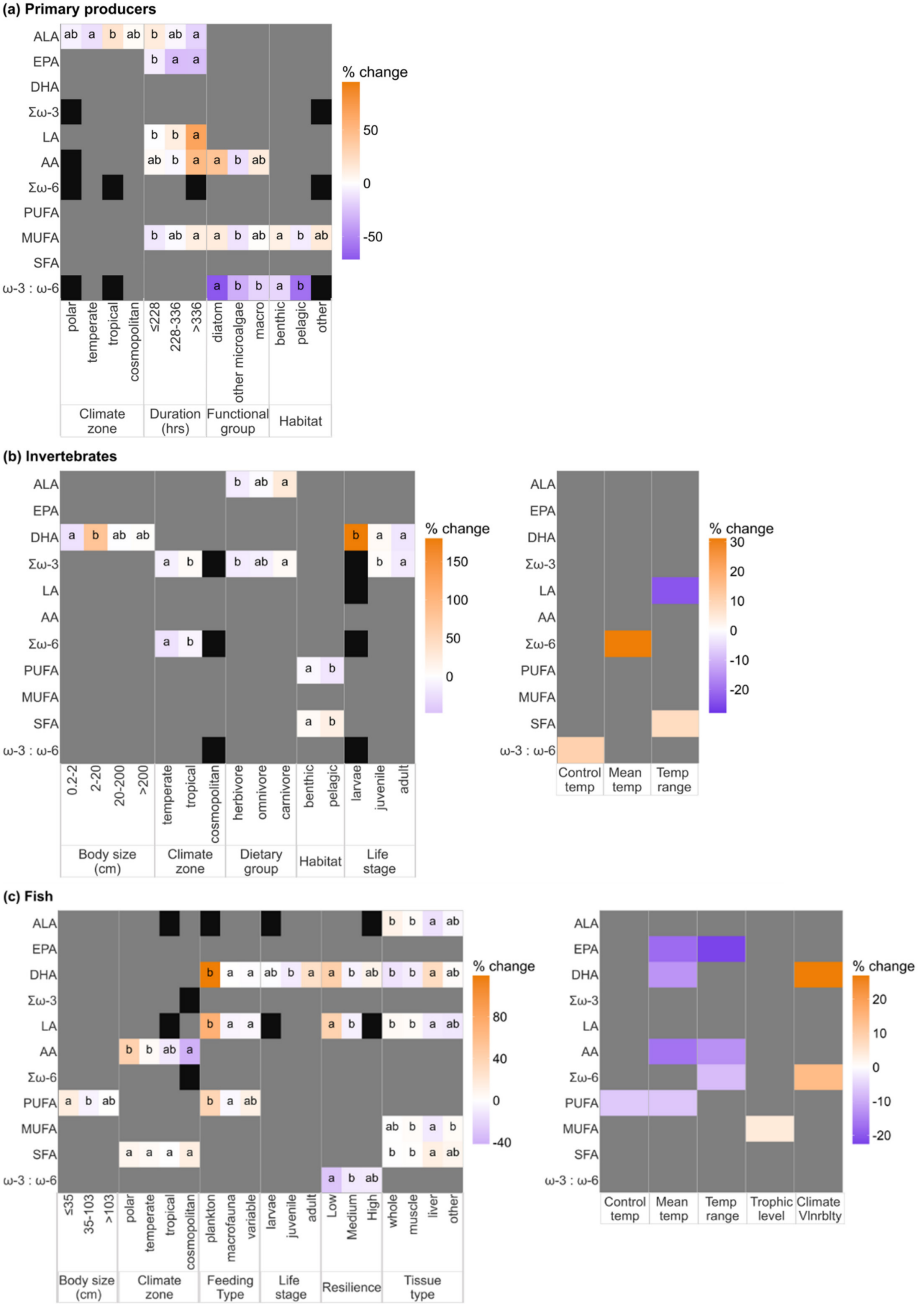
Heat maps showing variations in the effects of warming on FA proportions across moderators for (a) primary producers, (b) invertebrates and (c) fish. Rows represent FAs and columns represent moderators, with categorical moderators on the left and continuous moderators on the right. Only significant results are shown (*p* < 0.05; ANOVA for categorical moderators, slope [*β*] for continuous moderators), with grey cells indicating non‐significant effects and black cells indicating missing data. For categorical moderators, cells show the estimated FA warming effect (ln*RR*) for each subcategory: Orange indicates an increase, blue a decrease and white no change. Letters denote pairwise comparisons of estimated marginal means between subcategories from post hoc tests, with different letters indicating significantly different means (*p* < 0.05). For continuous moderators, cells show the slope (*β*) of the relationship between the FA warming effect (ln*RR*) and the moderator: Orange indicates positive relationships and blue negative. FA acronyms as per Figure 1.

Across all taxa groups, warming‐induced changes in FA proportions varied across biological traits and methodological moderators, often in opposing directions (Figure 3). In primary producers, warming effects on FA proportions varied significantly across climate zone origin, exposure duration, functional group and habitat type (Figure 3a; Table 1). Notably, omega‐3: omega‐6 ratios declined much more in diatoms (−71% ± 24.3) than in macro primary producers (−18% ± 16.9) or other microalgae (−34% ± 30.9) and more in pelagic primary producers (−61% ± 23.3) than in benthic primary producers (−17% ± 23.0) (Figure 3a; Table 1).

**TABLE 1:** Effects of warming on primary producer FA proportions (% total FAs) across moderators.

Moderator	Subcategory	FA response to warming (mean ± SE)
Climate zone	Temperate	ALA: −13% ± 6.4
	Tropical	ALA: +19% ± 10.3
Functional group	Diatom	AA: +43% ± 18.1 MUFA: +14% ± 6.2 *ω*‐3: *ω*‐6 ratio: −71% ± 24.3
	Other microalgae	AA: −14% ± 10.6 MUFA: −12% ± 5.2 *ω*‐3: *ω*‐6 ratio: −34% ± 30.9
	Macro primary producer	*ω*‐3: *ω*‐6 ratio: −18% ± 16.9
Habitat	Benthic	MUFA: +10% ± 6.7 *ω*‐3: *ω*‐6 ratio: −17% ± 23.0
	Pelagic	MUFA: −8% ± 4.3 *ω*‐3: *ω*‐6 ratio: −61% ± 23.3
Experiment duration (h)	≤ 228	ALA: +14% ± 7.9 EPA: −8% ± 6.4 MUFA: −10% ± 6.0 LA: −1% ± 10.2 AA: +4% ± 8.2
	228–336	EPA: −28% ± 7.4
	> 336	ALA: −19% ± 12.8 MUFA: +12% ± 8.0 LA: +67% ± 17.0 AA: +50% ± 17.6

*Note:* Only responses that differed significantly from at least one other subcategory for categorical moderators (post hoc pairwise comparisons of estimated marginal means; *p* < 0.05), or responses with significant slopes (*β*; *p* < 0.05) for continuous moderators are shown. Responses are expressed as the estimated warming effect on FA proportions (ln*RR*). FA acronyms as per Figure 1.

In invertebrates, proportions were significantly modulated by body size, climate zone origin, dietary group, habitat type, life stage, absolute control temperature, mean preferred temperature and preferred temperature range (Figure 3b; Table 2). In particular, DHA increased greatly under warming in invertebrates within the 2–20 mm size class (+81% ± 25.0) and in larvae (+180% ± 29.2) but decreased in those within the 0.2–2 mm size class (−23% ± 18.0) and in adults (−20% ± 12.6). Additionally, total omega‐3 declined more in temperate invertebrates (−12% ± 5.2) than tropical invertebrates (+5% ± 7.0), in herbivores (−15% ± 7.1) than carnivores (+7% ± 6.8) and in adults (−16% ± 5.8) than juveniles (+4% ± 5.7; no data for larvae). Pelagic taxa had greater declines in PUFA (−20% ± 5.9) and increases in SFA (+20% ± 3.0) than benthic taxa (PUFA: −6% ± 5.3; SFA: +7% ± 2.4) and SFA proportions increased more as the taxa's preferred temperature range increased (*β* = +8% ± 3.0). Omega‐3: omega‐6 ratios declined more strongly as control temperature decreased (*β* = +11% ± 4.7) (Figure 3b; Table 2).

**TABLE 2:** Effects of warming on invertebrate FA proportions (% total FAs) across moderators.

Moderator	Subcategory	FA response to warming (mean ± SE or slope *β*)
Body size (mm)	0.2–2	DHA: −23% ± 18.0
	2–20	DHA: +81% ± 25.0
Climate zone	Temperate	Total *ω*‐3: −12% ± 5.2 Total *ω*‐6: −23% ± 4.5
	Tropical	Total *ω*‐3: 5% ± 7.0 Total *ω*‐6: −8% ± 5.8
Diet group	Herbivore	ALA: −15% ± 11.0 Total *ω*‐3: −15% ± 7.1
	Carnivore	ALA: 29% ± 13.3 Total *ω*‐3: +7% ± 6.8
Habitat	Benthic	PUFA: −6% ± 5.3 SFA: +7% ± 2.4
	Pelagic	PUFA: −20% ± 5.9 SFA: +20% ± 3.0
Life stage	Larval	DHA: +180% ± 29.2
	Juvenile	Total *ω*‐3: +4% ± 5.7 DHA: +7% ± 13.5
	Adult	Total *ω*‐3: −16% ± 5.8 DHA: −20% ± 12.6
Mean preferred temperature	NA	Total *ω*‐6: *β* = +31% ± 7.9
Preferred temperature range	NA	SFA: *β* = +8% ± 3.0 LA: *β* = −24% ± 9.3
Control temperature	NA	*ω*‐3: *ω*‐6 ratio: *β* = +11% ± 4.7

*Note:* Only responses that differed significantly from at least one other subcategory for categorical moderators (post hoc pairwise comparisons of estimated marginal means; *p* < 0.05), and responses with significant slopes (*β*; *p* < 0.05) for continuous moderators are shown. For categorical moderators, responses are expressed as the estimated warming effect on FA proportions (ln*RR*). For continuous moderators, responses are the slope (*β*) of the relationship between the FA warming effect (ln*RR*) and the moderator. FA acronyms as per Figure 1.

In fish, eight biological traits and three methodological moderators modulated FA proportional responses to warming (Figure 3c; Table 3). Notably, warming induced very large DHA proportion increases in plankton feeders (+119% ± 33.7), but negligible changes in macrofauna predators (0% ± 7.9) and variable feeders (−1% ± 13.1). DHA also increased in adults (+28% ± 11.2) and low‐resilience species (+42% ± 16.6), contrasting with responses in juveniles (−10% ± 8.5) and medium‐resilience species (−6% ± 7.8). Omega‐3: omega‐6 ratios declined in low‐resilience species (−30% ± 6.7) but increased in medium‐resilience species (+12% ± 5.6; data for high‐resilience species were limited). DHA proportions increased more under warming as climate vulnerability increased (*β* = 27% ± 6.6). The liver showed increases in DHA (31% ± 12.5) and SFA (14% ± 2.9), in contrast to the whole body (DHA: −14% ± 12.4; SFA: 1% ± 2.7) and muscle tissue (DHA: −9% ± 12.5; SFA: 1% ± 2.7). Larger decreases in PUFA occurred as control temperatures increased (*β* = −6% ± 3.0), and decreases in EPA (*β* = −18% ± 9.1), DHA (*β* = −13% ± 6.9) and PUFA (*β* = −7% ± 3.4) became greater as mean preferred temperature increased. Larger decreases in EPA also occurred as the preferred temperature range increased (*β* = −22% ± 8.9) (Figure 3c; Table 3).

**TABLE 3:** Effects of warming on fish FA proportions (% total FAs) across moderators.

Moderator	Subcategory	FA response to warming (mean ± SE or slope *β*)
Body size (cm)	≤ 35	PUFA: +16% ± 7.0
	35–103	PUFA: −6% ± 5.0
Climate zone	Polar	AA: +41% ± 12.8
	Temperate	AA: +4% ± 6.8 SFA: +4% ± 2.2 (marginal difference *p* = 0.066)
	Cosmopolitan	AA: −41% ± 19.2 SFA: +15% ± 3.6 (marginal difference *p* = 0.066)
Feeding type	Plankton feeder	DHA: +119% ± 33.7 LA: +71% ± 22.2 PUFA: +37% ± 13.9
	Macrofauna predator	DHA: 0% ± 7.9 LA: −6% ± 7.2 PUFA: −4% ± 4.2
	Variable	DHA: −1% ± 13.1 LA: −4% ± 9.2
Life stage	Juvenile	DHA: −10% ± 8.5
	Adult	DHA: +28% ± 11.2
Mean preferred temperature	NA	EPA: *β* = −18% ± 9.1 DHA: *β* = −13% ± 6.9 AA: *β* = −17% ± 6.1 PUFA: *β* = −7% ± 3.4
Preferred temperature range	NA	EPA: *β* = −22% ± 8.9 AA: *β* = −13% ± 6.3 Total *ω*‐6: *β* = −8% ± 4.0
Resilience to fishing pressure	Low	DHA: +42% ± 16.6 LA: +40% ± 15.5 *ω*‐3: *ω*‐6: −30% ± 6.7
	Medium	DHA: −6% ± 7.8 LA: −8% ± 6.1 *ω*‐3: *ω*‐6: −12% ± 5.6
Trophic level	NA	MUFA: *β* = +4% ± 1.8
Vulnerability to climate change	NA	DHA: *β* = +27% ± 6.6 Total *ω*‐6: *β* = +14% ± 4.5
Control temperature	NA	PUFA: *β* = −6% ± 3.0
Tissue type	Whole body	ALA: +12% ± 8.5 LA: +6% ± 9.2 MUFA: 0% ± 4.1 DHA: −14% ± 12.4 SFA: +1% ± 2.7
	Muscle	ALA: +3% ± 8.1 LA: +3% ± 8.9 MUFA: +3% ± 3.9 DHA: −9% ± 12.5 SFA: +1% ± 2.7
	Liver	ALA: −16% ± 8.2 LA: −11% ± 9.3 MUFA: −11% ± 3.8 DHA: +31% ± 12.5 SFA: +14% ± 2.9

*Note:* Only responses that differed significantly from at least one other subcategory for categorical moderators (post hoc pairwise comparisons of estimated marginal means; *p* < 0.05), and responses with significant slopes (*β*; *p* < 0.05) for continuous moderators are shown. Responses are expressed as the estimated warming effect on FA proportions (ln*RR*). FA acronyms as per Figure 1.

FA concentration responses to warming across moderator levels were largely distinct from those for proportions, affecting different moderators and FA groups (Figure S8; Supplementary Results). In primary producers, polar species had large declines in FA concentrations (−38% to −90% across total FA, ALA, EPA, AA) compared to temperate, tropical or cosmopolitan species that generally showed neutral or positive responses (Figure S8a). Similarly, adult or stationary growth phases had larger concentration declines across ALA, DHA, total omega‐3 and PUFA (−19% to −23%) than immature or exponential growth phases (9% to 46%). Exposure duration influenced 7 out of 12 FA groups in varying ways and LA increased with higher control temperatures (*β* = 53% ± 21.6) (Figure S8a). In invertebrates, few moderators had significant effects; however, DHA largely increased in the 2–20 mm size class (193% ± 32.3) and the larval life stage (207% ± 57.7) in contrast to other size classes (−20% to −76%) and life stages (−20% to 25%). Intermediate durations (168–720 h) also drove larger decreases in total omega‐3 (−22% ± 69) than long exposures (> 720 h) (16% ± 14.7) (Figure S8b). In fish, larger decreases in total omega‐3, total omega‐6 and PUFA concentrations occurred in intermediate body sizes (35–103 cm) (−53% to −71%) than small (≤ 35 cm) (−22% to −27%) and large (> 103 cm) (−26% to −28%) body sizes (Figure S8c). Larger declines in total omega‐3, LA, total omega‐6 and PUFA also occurred with intermediate exposures (240–2920.8 h) (−46% to −71%) than short (≤ 240 h) (−23% to −25%) or long (> 2920.8 h) (−26% to 13%) exposures (Figure S8c; Supplementary Results).

Residual heterogeneity was significant in 627/643 models across proportion and concentration data and was very high (*I* ^2^_total_ > 80%) in 593/643. The distribution of true heterogeneity varied greatly across hierarchical random effect levels. 553/643 models explained ≥ 50% of variation in ln*RR* (*R* ^2^_*c*_) (Table S9; Supplementary Results).

## 4 Discussion

This study represents the first global meta‐analysis to assess the comprehensive impact of climate change stressors, specifically ocean warming and acidification, on fatty acid (FA) profiles across marine primary producers, invertebrates and fish. Our findings reveal clear taxon‐specific responses of FA proportions and concentrations, with warming emerging as the dominant driver, whilst acidification effects were generally smaller and less consistent. Critically, warming generally increased saturated fatty acid (SFAs) proportions and concentrations and reduced health‐critical polyunsaturated fatty acid (PUFA) proportions and concentrations, particularly long‐chain omega‐3 FAs such as EPA and DHA. These profound biochemical shifts resulted in significant declines in omega‐3: omega‐6 ratios, a key index of nutritional quality for both marine organisms and human consumers. These patterns were strongest and most consistent in primary producers across all warming levels, whilst strong proportional shifts in invertebrates were supported at high warming levels (> 10°C) alongside substantial reductions in omega‐3 and total FA concentrations. In contrast, fish exhibited fewer proportional changes but displayed the most consistent and strongly supported declines in concentrations across FA groups, suggesting reduced absolute nutrient availability, despite relatively stable FA compositions. Considering both FA proportions and concentrations revealed complementary insights on the nutritional quality and quantity, with both metrics negatively affected by warming. Collectively, these findings highlight significant implications for nutrient trophic transfer, fisheries productivity and food security under ocean warming.

The decline of omega‐3 PUFAs (including EPA and DHA) in primary producers is particularly concerning because marine algae are the main source of these important FAs globally (Hixson and Arts 2016). This decline directly limits omega‐3 availability to primary consumers and may cascade to higher trophic levels (Hixson and Arts 2016; Parrish 2025), impacting growth and development, reproduction, immune function and metabolic resilience (Ahlgren et al. 1994; Baird 2024; Ghinter et al. 2025). The threat of limited EPA and DHA availability is exacerbated by predictions of climate‐induced community shifts favouring cyanobacteria that lack long‐chain PUFA production and DHA‐poor cladocerans over PUFA‐producing diatoms and DHA‐rich copepods (Gladyshev et al. 2013). Declines of 34%–53% in omega‐3: omega‐6 ratios of primary producers across all warming levels may further reduce nutritional quality and nutrient transfer efficiency, with implications for population dynamics and community structure (Beca‐Carretero et al. 2018; McLaskey et al. 2019).

In fish, reduced total FA concentrations of up to 43% may also decrease energy yields available to predators, compounding the effects of nutritional quality (Barbosa et al. 2017). For humans, who convert ALA to EPA and DHA inefficiently (Pilecky et al. 2021), declines in marine omega‐3 availability may exacerbate already widespread dietary sub‐optimal levels (Brett et al. 2006; Elsdon 2010). Declining omega‐3: omega‐6 ratios raise further concerns, given many diets globally are disproportionately high in omega‐6 FAs (Hixson and Arts 2016). An increasing shift towards omega‐6 dominance in seafood may further skew omega‐3: omega‐6 ratios, elevating risks of immune and cardiovascular diseases (Simopoulos 2002). Furthermore, reduced total FA concentrations may also lower energy yields derived from fisheries that are already threatened by multiple pressures, including overexploitation and habitat degradation. Although aquaculture may partially mitigate these effects, current feed sources largely rely on wild‐caught fish and plant oils rich in omega‐6, making it increasingly challenging to maintain sufficient omega‐3 levels under warming conditions (Oliver et al. 2020; Pilecky et al. 2021).

Overall increases of SFAs due to warming and corresponding PUFA reductions are consistent with membrane lipid remodelling via homeoviscous adaptation, whereby organisms adjust membrane FA composition to maintain fluidity under changing temperatures, a process that has been demonstrated previously across primary producers, invertebrates and fish (Fuschino et al. 2011; Sharma et al. 2012; Valles‐Regino et al. 2015). Maintaining appropriate membrane lipid order helps preserve protein structure and activity, ion gradients and signal transduction that underpin key physiological processes such as respiration, growth and reproduction (Betancor et al. 2012; Guschina and Harwood 2006; Sharma et al. 2012). The mechanisms underlying this process likely vary among taxa and may involve redistribution of FAs from storage or other tissues, selective dietary incorporation of SFAs over PUFAs and downregulation of desaturase enzymes that synthesise PUFAs (Bogevik et al. 2011; Guschina and Harwood 2006). Patterns in primary producers and invertebrates are largely consistent with these mechanisms, but in fish, despite slight increases in SFA proportions, PUFA proportions showed limited declines. However, the observed reductions in MUFA proportions in fish possibly suggest MUFAs role as substitutes for PUFAs in maintaining membrane fluidity, corroborating previous studies (Brodte et al. 2008; De Santis et al. 2018).

Notable shifts in omega‐3 and omega‐6 dynamics provide insights into the biochemical adjustments that underlie these trends. Across taxa, warming generally decreased omega‐3: omega‐6 ratios, with the largest reductions occurring under higher warming. In primary producers, proportions of EPA, DHA and total omega‐3 FAs decreased, whilst omega‐6 proportions tended to increase in agreement with previous studies (Hixson and Arts 2016). Although confidence intervals for total omega‐6 FA increases overlapped zero, this was only marginal under high warming and the limited number of effect sizes reduced the statistical power. The opposing responses of omega‐3 and omega‐6 FAs reflect their shared enzymatic pathways for synthesis and catabolism, whereby enhancing the production or breakdown of one group inherently suppresses the other (Ahlgren et al. 1994). Omega‐3 pathway suppression could explain the increase in ALA at medium warming levels in primary producers alongside decreases in EPA and DHA, whereby dietary ALA may be converted less into longer chain omega‐3 FAs. For taxa with limited ability for long‐chain PUFA synthesis, such as most marine fish and some invertebrates, this omega‐3: omega‐6 ratio shift may instead reflect the greater oxidative susceptibility of omega‐3 FAs due to their generally higher degree of unsaturation (Betancor et al. 2012; Rodriguez‐Amaya and Shahidi 2021), coupled with enhanced catabolism or peroxidation under thermal stress (Chokshi et al. 2015). An exception occurred among invertebrates at the highest warming level, where omega‐6 proportions declined more than omega‐3 proportions, resulting in an increase in omega‐3: omega‐6 ratio (with marginal support). This result may reflect extreme metabolic demands under thermal stress, leading to large increases in peroxidation across PUFA groups (Chokshi et al. 2015), also reflected by the significant reduction in total FA concentrations. However, limited availability across concentration data for invertebrates at high warming levels constrains the interpretation of the underlying mechanisms.

Distinct taxonomic differences emerged in total FA concentration responses to warming. In primary producers, total FAs remained relatively stable or even increased under moderate warming, possibly due to thermal stress‐induced redirection of carbon away from growth and reproductive processes towards storage lipid accumulation (Feijão et al. 2018; Suparmaniam et al. 2023). In contrast, total FA concentrations in invertebrates and fish declined with increasing temperature, likely due to increased FA catabolism in response to higher energy requirements (Fernandes et al. 2021; Lischka et al. 2022). Reduced lipid anabolism may also contribute to lower FA levels in some taxa, as warming can decrease mitochondrial densities which limit energy and metabolic resources for biosynthesis (Brodte et al. 2008). Under more extreme stress, FA reserves may be further depleted (Fernandes et al. 2021), consistent with the pronounced declines observed in fish across all FA concentrations, including a 50% reduction in DHA under high warming. These declines suggest that warming may reduce the overall nutrient availability to higher consumers, even if compositional ratios remain relatively stable. However, despite the implications of declining EPA and DHA availability appearing largely negative in terms of nutritional quality across marine food webs and human diets, the implications may vary among ecosystems and taxa. Species with high dietary dependence on long‐chain omega‐3 FAs, particularly during vulnerable life stages, may be disproportionately affected by reduced availability whilst other species may partially compensate through physiological adjustments, selective retention of important FAs or shifts in dietary intake.

In contrast to warming, FA responses to acidification were typically weakly supported, more variable across FA groups and taxa and often small in magnitude, rarely exceeding changes of ±20%. Acidification analyses, particularly for concentrations, were constrained by smaller sample sizes, which may have contributed to these weaker effects. Concentration data showed a few stronger responses to acidification such as declines in total FA and DHA in primary producers and increases in PUFA and EPA in invertebrates, indicating possible evidence of shifts under acidification. The overall weaker responses may be partly due to limited data, but they also align with previous findings that temperature exerts stronger biochemical influence than acidification (Garzke et al. 2017). Further studies should investigate the impacts of acidification, particularly alongside warming, to capture whether interactive effects on marine FAs may amplify, or possibly counteract, shifts.

Our models revealed several effects of biological trait and methodological moderators that were otherwise obscured in the pooled analyses. Diatoms showed strong increases in AA proportions alongside large declines of 71% in omega‐3: omega‐6 ratios, suggesting larger shifts towards omega‐6 synthesis under warming compared to other primary producers. MUFA proportions also increased in diatoms, benthic primary producers and high trophic level fish, but decreased in other microalgae, pelagic primary producers and low trophic level fish, suggesting diverse remodelling strategies where MUFAs may act as a substitute for either SFAs or PUFAs (Hixson and Arts 2016; Fuschino et al. 2011).

Species from environments with broader temperature ranges generally showed stronger evidence of homeoviscous adaptation (characterised by decreased PUFA and increased SFA levels) than those from more thermally stable environments. This is perhaps due to stronger selective pressure for thermal resilience through physiological shifts that result in greater FA composition plasticity (Brodte et al. 2008; Peck et al. 2014; Twining et al. 2021). For example, cosmopolitan and temperate taxa that are typically exposed to wide thermal variability displayed larger FA proportional shifts than tropical and polar species, which have evolved in more thermally stable environments (Peck et al. 2014; Sunday et al. 2011). A negative correlation with control temperature suggested that temperate fish exhibited larger PUFA proportion losses than polar fish, given the limited tropical fish data. For invertebrates, polar data were limited, meaning that the positive correlation with preferred mean temperature suggested that temperate invertebrates displayed larger losses in omega‐6 proportions than tropical invertebrates. Pelagic invertebrates, exposed to greater thermal variations from surface/mixed‐layer dynamics and diel vertical migrations than benthic taxa from the more stable seafloor, also showed larger PUFA proportion reductions and SFA increases than benthic taxa (Brierley 2014; Wilson et al. 2025). Similarly, species from more thermally variable environments displayed larger omega‐3: omega‐6 ratio declines which may reflect their stronger responses to thermal change (Peck et al. 2014). Accordingly, larger omega‐3: omega‐6 ratio declines occurred in pelagic primary producers and temperate invertebrates (inferred from control temperature, given limited polar data for this FA group) than benthic and tropical taxa. Omega‐6 concentration gains were also larger in temperate primary producers than polar (inferred from control temperature, given limited tropical data for this FA group).

Metabolic changes or constraints may also influence FA responses. Tropical primary producers exhibited ALA proportion increases, possibly because high baseline temperatures may constrain desaturase activity further if already near their upper limit (Nguyen et al. 2011), limiting conversion of ALA to long‐chain PUFAs. Smaller fish (≤ 35 cm), which tend to grow rapidly, displayed increases in PUFA proportions, possibly due to prioritising PUFA retention for sustaining fast growth (Márquez et al. 2024), whilst 35–103 cm fish showed PUFA proportion declines, although limited sample size and taxonomic biases (small body size category was dominated by polar taxa, and medium by cosmopolitan and temperate taxa) make this explanation uncertain. Amongst invertebrate larvae, the approximately three‐fold increase in DHA proportions and concentrations may also arise from warming‐driven acceleration of growth, which enhances demand for DHA (Figueiredo et al. 2009; Gladyshev et al. 2018), a key micronutrient for growth and development (Garzke et al. 2017; Tocher 2010). Additionally, similarly large increases observed amongst 2–20 mm invertebrates may be a consequence of sampling bias, as 80% of samples in this size class were larvae. In fish, DHA proportion increases in adults may be a result of DHA conservation for other processes such as reproduction (Trushenski and Rombenso 2020), whilst DHA decreases in juvenile fish may be because utilisation rates exceed replenishment rates under elevated growth and thermal stress (Mejri et al. 2021). In primary producers, the immature or exponential phase maintained or increased PUFA and omega‐3 concentrations, whereas the adult or stationary phase showed reductions, possibly reflecting slower growth rate and reduced priority in retaining those FAs in the latter group. Stationary stages may also shift towards carbohydrate accumulation over lipid, reducing FA replenishment after catabolism (Boelen et al. 2017; El‐Haji et al. 2023).

Feeding behaviour was another ecological factor explaining variation in FA responses. Whilst overall declines in PUFAs were consistent with the theory of homeoviscous adaptation, we observed important exceptions related to diet. Notably, DHA proportions more than doubled in plankton feeders, such as plankton feeding fish and invertebrate larvae. This may be due to elevated metabolism increasing ingestion rates of filter feeders, leading to increased incorporation of these FAs relative to macrofauna predators where ingestion rate is more constrained by prey availability (Figueiredo et al. 2009; Helenius et al. 2020). Further work is needed to investigate whether filter feeding organisms could therefore play a role in offsetting broader declines observed across food webs. Dietary group also influenced invertebrate FA responses as ALA and total omega‐3 proportions declined in herbivores but increased in carnivores. Because FA composition reflects dietary sources (Brett et al. 2006; Torno et al. 2018), these differences may result from faster homeoviscous FA adjustments in live algal diets of herbivores than in the prey of carnivores (Sayanova et al. 2017).

Across fish, DHA proportion increases were also observed in low‐resilience, climate change vulnerable, polar (inferred from preferred mean temperature) and liver tissue, all of which are associated with high sensitivity to thermal physiological stress (Peck et al. 2014; Pineda et al. 2012; Liu et al. 2022). Despite DHA being highly susceptible to peroxidation due to its very high unsaturation index (Betancor et al. 2012), it may also stimulate antioxidant enzyme activity which provides protection against oxidative damage (Fernandes et al. 2021), highlighting its complex physiological roles across species and contexts (Brodte et al. 2008; Holm et al. 2022). Increases in DHA, not only in these fish groups but also in invertebrate larvae, may therefore reflect a compensatory mechanism to enhance cellular protection under oxidative or thermal stress (Sanchez‐Valle et al. 2012; Qiao et al. 2016).

Fish with lower resilience to climate change displayed larger reductions in omega‐3: omega‐6 ratios. This may occur because greater oxidative stress in sensitive taxa could accelerate FA degradation, widening the gap between omega‐3 and omega‐6 contents faster than in less sensitive taxa due to the higher peroxidation susceptibility of omega‐3 FAs (Betancor et al. 2012; Rodriguez‐Amaya and Shahidi 2021). Both polar and adult or stationary phase primary producers showed larger and more consistent FA concentration losses than other subgroups, perhaps due to high rates of warming‐induced oxidative damage degrading FAs (Chokshi et al. 2015). Stationary phase organisms are highly sensitive to physiological stress because they often experience high rates of nutrient limitation, crowding and cumulative oxidative damage, which may be further exacerbated under elevated temperatures (Chokshi et al. 2015). Alternatively, the larger FA concentration losses may also reflect high baseline levels of lipid reserves in polar and adult or stationary phase primary producers (Brodte et al. 2008; Boelen et al. 2017). Taxa with larger lipid stores may rely more heavily on these reserves to meet raised metabolic demands under warming, resulting in higher rates of FA depletion.

Exposure duration also influenced FA responses and suggests temporal plasticity in some taxa groups. In primary producers, effects often intensified with longer exposure, whereas in invertebrates and fish, no changes to FA proportions occurred but PUFA concentration declines were strongest at medium durations, although small sample sizes increased uncertainty. Short exposures may not allow much physiological adjustment, whereas long exposures could allow acclimation (Chokshi et al. 2015). Differences in the effects of duration among taxa groups may reflect variation in experimental durations, with ‘long’ exposures only exceeding two weeks for primary producers, one month for invertebrates and four months for fish. Given ocean warming describes chronic, long‐term exposure to elevated temperatures, longer experimental durations may better approximate long‐term climate change outcomes in marine environments, whereas short durations are more informative for assessing short‐term heatwave effects.

Tissue‐specific responses in fish occurred. Liver tissue, central to lipid synthesis, storage and FA export, showed declines in ALA, LA and MUFA proportions, whilst long‐chain PUFA was generally preferentially retained, perhaps for critical physiological functions such as inflammatory responses and reproductive roles (Chee et al. 2020; Sayyaf Dezfuli et al. 2023; Sun et al. 2023). In contrast, muscle and whole‐body tissues maintained or increased ALA and LA proportions, suggesting redistribution of these FAs from liver to peripheral tissues, perhaps to fuel elevated metabolic demands.

Several limitations constrain the interpretation of these results. All studies were controlled experiments, which may not fully capture the interactive and additive effects of multi‐stressors in natural environments (Fuschino et al. 2011). Furthermore, experimental conditions varied widely, including diet, salinity and light regimes, which may have interacted with temperature and pH effects (Becker et al. 2010; Brett et al. 2006; Khériji et al. 2003). Most experiments were short (< 1 month), limiting inferences about long‐term acclimation or evolutionary adaptation, although short‐term responses remain ecologically relevant given the rising frequency of marine heatwaves (IPCC 2023; Oliver et al. 2018). Normalising exposure duration by generation time could help distinguish short‐term physiological responses from acclimation, but generation time data were unavailable for many species. Many experiments applied unrealistic temperature treatments (e.g., cooling or extreme warming) to address a variety of research objectives. Another source of uncertainty is dietary exposure to environmental treatments. Most fish studies used non‐living feed, making temperature and acidification effects mediated through diet unlikely. In contrast, approximately half of the invertebrate studies used live food that may also have been exposed to the treatments, potentially contributing to observed FA changes. However, prey exposure conditions were generally not reported, preventing an analysis without substantial assumptions. Inconsistencies in FA reporting (units, tissues, lipid classes and FA groups) further reduced comparability across studies, and data gaps were evident, especially for FA concentrations, acidification and tropical fish. Lastly, unavailable biological trait data, especially for primary producers and invertebrates, limited the moderators that could be assessed.

## 5 Conclusions

This meta‐analysis demonstrates that ocean warming is a pervasive stressor consistently leading to systemic reduction of omega‐3 FA proportions and concentrations and omega‐3: omega‐6 ratios across marine taxa, whilst increasing SFAs. These detrimental biochemical alterations, especially at the base of the food chain, fundamentally threaten the nutritional quality of marine resources, thereby undermining the ocean's capacity to supply long‐chain omega‐3 FAs required for both marine biodiversity, fisheries productivity and human nutrition (McLaskey et al. 2019). Our study found warming to be a stronger driver of these nutritional shifts than acidification, although future research should further explore the potential synergistic effects of combined stressors. Furthermore, the strong influence of biological traits (e.g., climate zone, life stage) and methodological moderators (e.g., exposure duration) on FA responses underscores the necessity of moving beyond general predictions. Future research should expand to understudied taxa, life stages and regions, particularly to the rapidly warming tropics that often support human populations already experiencing micronutrient deficiencies (Brown et al. 2022; Robinson et al. 2022). Anticipating and mitigating these climate‐driven nutritional declines is a critical imperative for effective conservation, fisheries management and aquaculture strategies to secure global food security and maintain ecological resilience.

## Author Contributions

**Sean D. Connell:** conceptualization, writing – review and editing, visualization, supervision. **Bethany Rose Dawson:** conceptualization, methodology, formal analysis, investigation, data curation, writing – original draft, visualization, project administration. **Evangeline Mantzioris:** conceptualization, writing – review and editing, visualization, supervision. **Camille Mellin:** conceptualization, methodology, validation, resources, writing – review and editing, visualization, supervision, project administration, funding acquisition. **Ivan Nagelkerken:** conceptualization, writing – review and editing, visualization, supervision.

## Funding

This work was supported by the Australian Research Council (Grants DP230101932 and FT200100870).

## Conflicts of Interest

The authors declare no conflicts of interest.

## Supporting information


**Data S1:** Methods.

**Data S2:** Results.

**Figure S1:** PRISMA flow diagram depicting the number of studies identified in the literature search and then included or excluded during each stage of screening. The total number of studies is represented by n, whilst values in parentheses following n indicate concentration (Conc) and proportion (Prop) counts, respectively.
**Figure S2:** Histograms showing the distribution of (a) maximum body size across fish; and (b) the duration across primary producers, invertebrates and fish. Dashed vertical lines indicate the subcategory boundaries as defined based on the data distribution.
**Figure S3:** Funnel plots of effect sizes for (a) warming and (b) acidification, plotted against precision (inverse of SE) for each FA group across primary producers, invertebrates and fish. Dashed vertical lines indicate the mean effect size.
**Figure S4:** Histograms for (a) warming and (b) acidification showing the distribution of log response ratio (ln*RR*) values for each FA group across primary producers, invertebrates and fish.
**Figure S5:** Histograms showing the distribution of (a) warming and (b) acidification increase values across primary producers, invertebrates and fish. Dashed vertical lines indicate the subcategory boundaries as defined based on the data distribution, delineating low, medium and high levels respectively.
**Figure S6:** Percentage changes in FA absolute concentration response to three warming levels (≤ 6°C, 6°C–10°C and > 10°C) calculated from average log response ratio (ln*RR*) slope coefficient estimates (*β*). The thick, medium and narrow horizontal bars denote 85%, 90% and 95% confidence intervals, respectively, and the dashed vertical line represents a null effect. Filled circles are where 85% confidence intervals do not overlap zero. Estimates to the right of zero indicate increases under warming, whilst those to the left indicate decreases. Results were considered to have very strong, strong and moderate support at the 95%, 90% and 85% confidence levels, respectively. Open circles show effects whose 85% confidence intervals overlap zero, indicating no consistent directional effect. Species represented across all models: primary producers (*n* = 45), invertebrates (*n* = 15), fish (*n* = 9). FA acronyms as per Figure 1.
**Figure S7:** Percentage changes in FA absolute concentrations in response to three acidification levels (≤ 582, 582–1700 and > 1700 μatm) calculated from average log response ratio (ln*RR*) slope coefficient estimates (*β*). The thick, medium and narrow horizontal bars denote 85%, 90% and 95% confidence intervals, respectively, and the dashed vertical line represents a null effect. Estimates to the right of zero indicate increases under warming, whilst those to the left indicate decreases. Results were considered to have very strong, strong and moderate support at the 95%, 90% and 85% confidence levels, respectively. Open circles show effects whose 85% confidence intervals overlap zero, indicating no consistent directional effect. Species represented across all models: primary producers (*n* = 15), invertebrates (*n* = 4), fish (*n* = 3). FA acronyms as per Figure 1.
**Figure S8:** Heat maps showing the effects of warming on FA concentrations for (a) primary producers, (b) invertebrates and (c) fish. Rows represent FAs and columns represent moderators, with categorical moderators on the left and continuous moderators on the right. Only significant results are shown (*p* < 0.05; ANOVA for categorical moderators, slope [*β*] for continuous moderators), with grey cells indicating non‐significant effects and black cells indicating missing data. For categorical moderators, cells show the estimated FA warming effect (ln*RR*) for each subcategory: orange indicates an increase, blue a decrease and white no change. Letters denote pairwise comparisons, with different letters indicating significant differences (*p* < 0.05). For continuous moderators, cells show the slope (*β*) of the relationship between the FA warming effect (ln*RR*) and the moderator: orange indicates positive relationships and blue negative. FA acronyms as per Figure 1.

**Table S1:** Variables assessed as moderators of warming effects on FA levels with their subcategories for three taxa groups: primary producers, invertebrates and fish. Exclusion justifications and notes are indicated by superscripts: (1) insufficient data; (2) not informative; (3) highly unbalanced subcategories; (4) included, but with a largely reduced dataset.
**Table S2:** Results of Egger's test examining the relationship between effect sizes and effective sample sizes for temperature. NA values indicate cases where Egger's test could not be conducted due to insufficient variation or number of effective sample sizes.
**Table S3:** Species included across analyses. OA, ocean acidification; OW, ocean warming. Numbers in square brackets indicate how many studies included a specimen belonging to the specified genus that was not identified to the species level.
**Table S4:** The number of effect sizes (*k*), with the number of papers and experiments in parenthesis, included in each warming model for primary producers, invertebrates and fish.
**Table S5:** The number of effect sizes (*k*), with the number of papers and experiments in parenthesis, included in each acidification model for primary producers, invertebrates and fish.
**Table S6:** The number of effect sizes (*k*) within each subcategory included in each model for primary producers, invertebrates and fish.
**Table S7:** Heterogeneity and model summary statistics for warming effects on FA proportions and concentrations in primary producers, invertebrates and fish. QE is the *Q* statistic for residual heterogeneity (*Q* statistic), and QM is the Q statistic for the test of moderators (warming level), with significance indicated by asterisks (*p < 0.05; **p < 0.01; ***p < 0.001). *I* ^2^ values indicate the percentage of total variation in effect sizes (overall or across papers, taxa, experiments and effect sizes) that is attributable to true heterogeneity rather than sampling error. Marginal (*R* ^2^_*m*_) and conditional (*R* ^2^_*m*_) *R* ^2^ values are shown.
**Table S8:** Heterogeneity and model summary statistics for acidification effects on FA proportions and concentrations in primary producers, invertebrates and fish. QE is the *Q* statistic for residual heterogeneity (*Q* statistic), and QM is the *Q* statistic for the test of moderators (acidification level), with significance indicated by asterisks (*p < 0.05; **p < 0.01; ***p < 0.001). *I* ^2^ values indicate the percentage of total variation in effect sizes (overall or across papers, taxa, experiments and effect sizes) that is attributable to true heterogeneity rather than sampling error. Marginal (*R* ^2^_*m*_) and conditional (*R* ^2^_*m*_) *R* ^2^ values are shown.
**Table S9:** Heterogeneity and model summary statistics for the effects of warming on FA proportions and concentrations in primary producers, invertebrates and fish, with additional biological or experimental covariates included. QE is the *Q* statistic for residual heterogeneity (*Q* statistic), and QM is the *Q* statistic for the test of moderators (acidification level), with significance indicated by asterisks (*p < 0.05; **p < 0.01; ***p < 0.001). *I* ^2^ values indicate the percentage of total variation in effect sizes (overall or across papers, taxa, experiments and effect sizes) that is attributable to true heterogeneity rather than sampling error. Marginal (*R* ^2^_*m*_) and conditional (*R* ^2^_*m*_) *R* ^2^ values are shown. Rows in bold indicate significant moderator effects (*p* < 0.05; ANOVA for categorical, estimate/slope *β* for continuous).

**Data S3:** References.

## Data Availability

Data and R code for this manuscript, along with the bibliography of all accepted studies retained after screening, are available online at 10.6084/m9.figshare.31323259—https://figshare.com/s/b82eb890c6cf6b2f3e14.
