# Supplementary material for: The Effects of Ocean Warming and Acidification on Fatty Acid Contents of Marine Organisms: A Global Meta‐Analysis
Source: Glob Chang Biol. 2026 Aug 19;32(8):e71056. doi: 10.1111/gcb.71056 (PMC13487444; doi:10.1111/gcb.71056)
Supplement: Supplementary file 1 [file GCB-32-e71056-s001.docx]

**Supplementary Methods**

**Omega group assumptions**

When a study did not explicitly state the omega group classification of a FA but reported the carbon chain length and number of double bonds (e.g., 20:5), we made the following assumptions based on common FAs in biological samples and analytical FA methyl ester standards (e.g. Supelco 37 Component FAME Mix) used in chromatographic identification: (*i*) 20:5 was classified as EPA (eicosapentaenoic acid, omega-3), (*ii*) 22:6 as DHA (docosahexaenoic acid, omega-3), (*iii*) 18:2 as LA (linoleic acid, omega-6), and (*iv*) 20:4 as AA (arachidonic acid, omega-6). We did not assume 18:3 to be ALA (α-linolenic acid, omega-3), as multiple 18:3 isomers are common in biological samples and analytical FA methyl ester standards, and omega class cannot confidently be assigned without explicit reporting.

**Missing data**

Missing SDs were calculated using given SEs and sample sizes, and missing concentration data were calculated from proportion data, and vice versa, when total FA concentrations were provided (Equation S1). If the proportion of either PUFA, MUFA or SFA was missing, they were calculated from the other two, assuming the total must equal 100%. Total FAs were calculated from the sum of PUFA, MUFA, and SFA concentrations. Missing omega-3: omega-6 ratios were calculated when both omega-3 FA and omega-6 FA amounts (either proportion or concentration) were available, and the inverse of omega-6: omega-3 ratios was calculated.

Equation S1:

$$FA concentration=Total FA concentration\left( \frac{FA proportion}{100} \right)$$

Data for each FA were treated separately for imputing values. Sample sizes were imputed using the mean of reported sample sizes, after confirming the absence of a relationship between sample size and effect size (P > 0.05) (Kambach et al., 2020). For imputing SDs, the coefficient of variation (CV; %) was calculated for each row (Equation S2), and then the mean CV across all rows was determined and used for imputing SDs (Equation S3) (Nakagawa et al., 2023b). All SDs reported as 0 were also imputed using the same calculation. Samples sizes were imputed for 2.8% of data points and SDs were imputed for 33.2% of data points.

Equation S2:

$$CV= \frac{{SD}_{C}+{SD}_{T}}{{FA amount}_{C}+{FA amount}_{T}}$$

Equation S3:

$${SD}_{imp}= FA amount*mean CV$$

Where C = Control group; T = Treatment group; imp = imputation

**Data collection**

Due to the unbalanced number of data points in each body size class for fish, these were redefined into 3 subcategories based on the distribution of maximum adult body size data to ensure roughly equal sample sizes within groups (Figure S2).

For climate zone, taxa were assigned to one of four regions: tropical, temperate, polar, or cosmopolitan. The cosmopolitan category was assigned either when explicitly stated or when taxa spanned more than one climate zone. If the climate zone was not specified in the primary study, the geographic origin, which was identified as either the location where individuals were collected as stated in the primary study, or the original location of the aquaculture or laboratory stock, was used as a proxy: taxa from 30°N to 30°S were classified as tropical, 30-60°N and S as temperate, and 60-90°N and S as polar. The geographic origin of the taxa was recorded. Coordinates were obtained using Google Earth (Google LLC, 2023) when only location name was given.

To enable comparisons of life stage across macro and micro primary producers, life stage was defined as: stage 1 (larval or lag phase), stage 2 (immature or log phase), stage 3 (adult or stationary phase), and stage 4 (decay phase). For fish, invertebrates, and macro primary producers, adults were assumed when the life stage was not specified. For microalgae, growth curves published in the same study were used where available to estimate the growth phase at the time of sampling, otherwise life stage was left blank.

The preferred temperature range was calculated as the difference between the preferred maximum and minimum temperatures, using data available from SealifeBase (Palomares & Pauly, 2025) and FishBase (Froese & Pauly, 2023).

All acidification treatments reported in kPa were converted into µatm (Equation S4), whilst ppmv were treated as directly comparable to µatm. Studies only using pH units were excluded due to lack of comparability.

Equation S4:

$$\mu atm =kPa * \frac{{10}^{6}}{101.325}$$

**Model robustness checks and diagnostics**

Publication bias was tested using (1) funnel plots in which the effect sizes were plotted against the precision (inverse of SE) and (2) Egger’s tests examining the relationship between effect sizes and effective sample sizes (Nakagawa et al., 2022, 2023c). No strong evidence of publication bias was found as the funnel plots showed the effect size variation symmetrically decreasing as the precision increased, and Egger’s tests found no significant correlations between effect sizes and effective sample sizes except in some rare instances (Warming: fish total omega-3 concentration; Acidification: fish EPA proportion, invertebrate LA and total omega-6 proportion; p<0.05; Figure S3; Table S2).

Sensitivity analyses were undertaken to assess the robustness of results to potential influential studies that may sway the analysis (Nakagawa et al., 2023c). Each study was systematically removed in turn, and a series of meta-analytic models were run (“leave-one-out” method). No evidence of significant influence was found as the overall effect estimate of the full model fell within 95% confidence intervals (CIs) of each leave-one-out model (Nakagawa et al., 2023c) (Figure S4).

Residual heterogeneity was assessed using QE tests, where P < 0.05 indicates that the variability not explained by the moderators exceeds what would be expected from sampling error alone (Viechtbauer, 2010). The I^2^ statistic was calculated using the “orchaRd" *R* package (Nakagawa et al., 2023a) to estimate the proportion of total variability in effect size attributable to true heterogeneity, not sampling error (Nakagawa & Santos, 2012). Larger *I*^2^ values indicate that a greater proportion of the remaining variation after accounting for moderators reflects true heterogeneity rather than sampling error.
