# Supplementary material for: The Effects of Ocean Warming and Acidification on Fatty Acid Contents of Marine Organisms: A Global Meta‐Analysis
Source: Glob Chang Biol. 2026 Aug 19;32(8):e71056. doi: 10.1111/gcb.71056 (PMC13487444; doi:10.1111/gcb.71056)
Supplement: Supplementary file 2 [file GCB-32-e71056-s002.docx]

**Supporting Information References**

Froese, R., and D. Pauly. 2023. *Fish Base*. Version (02/2023). [www.fishbase.org](http://www.fishbase.org/).

Google LLC. 2023. *Google Earth*. [https://earth.google.com](https://earth.google.com/).

Kambach, S., H. Bruelheide, K. Gerstner, J. Gurevitch, M. Beckmann, and R. Seppelt. 2020. “Consequences of Multiple Imputation of Missing Standard Deviations and Sample Sizes in Meta‐Analysis.” *Ecology and Evolution* 10, no. 20: 11699–11712. <https://doi.org/10.1002/ece3.6806>.

Kambach, S., H. Bruelheide, K. Gerstner, J. Gurevitch, M. Beckmann, and R. Seppelt. 2020. “Consequences of Multiple Imputation of Missing Standard Deviations and Sample Sizes in Meta‐Analysis.” *Ecology and Evolution* 10, no. 20: 11699–11712. <https://doi.org/10.1002/ece3.6806>.

Nakagawa, S., M. Lagisz, M. D. Jennions, et al. 2022. “Methods for Testing Publication Bias in Ecological and Evolutionary Meta‐Analyses.” *Methods in Ecology and Evolution* 13, no. 1: 4–21. <https://doi.org/10.1111/2041-210X.13724>.

Nakagawa, S., M. Lagisz, R. E. O'Dea, et al. 2023a. “orchaRd 2.0: An R Package for Visualising Meta‐Analyses With Orchard Plots.” *Methods in Ecology and Evolution* 14, no. 8: 2003–2010. <https://doi.org/10.1111/2041-210X.14152>.

Nakagawa, S., D. W. A. Noble, M. Lagisz, R. Spake, W. Viechtbauer, and A. M. Senior. 2023b. “A Robust and Readily Implementable Method for the Meta‐Analysis of Response Ratios With and Without Missing Standard Deviations.” *Ecology Letters* 26, no. 2: 232–244. <https://doi.org/10.1111/ele.14144>.

Nakagawa, S., and E. S. A. Santos. 2012. “Methodological Issues and Advances in Biological Meta‐Analysis.” *Evolutionary Ecology* 26, no. 5: 1253–1274. <https://doi.org/10.1007/s10682-012-9555-5>.

Nakagawa, S., Y. Yang, E. L. Macartney, R. Spake, and M. Lagisz. 2023c. “Quantitative Evidence Synthesis: A Practical Guide on Meta‐Analysis, Meta‐Regression, and Publication Bias Tests for Environmental Sciences.” *Environmental Evidence* 12, no. 1: 8. <https://doi.org/10.1186/s13750-023-00301-6>.

Palomares, M. L. D., and D. Pauly. 2025. *Sea Life Base*. Version (04/2025). [www.sealifebase.org](http://www.sealifebase.org/).

Senior, A. M., C. E. Grueber, T. Kamiya, et al. 2016. “Heterogeneity in Ecological and Evolutionary Meta‐Analyses: Its Magnitude and Implications.” *Ecology* 97, no. 12: 3293–3299. <https://doi.org/10.1002/ecy.1591>.

Viechtbauer, W. 2010. “Conducting Meta‐Analyses in R With the Metafor Package.” *Journal of Statistical Software* 36, no. 3. <https://doi.org/10.18637/jss.v036.i03>.
