# Supplementary material for: The Effects of Ocean Warming and Acidification on Fatty Acid Contents of Marine Organisms: A Global Meta‐Analysis
Source: Glob Chang Biol. 2026 Aug 19;32(8):e71056. doi: 10.1111/gcb.71056 (PMC13487444; doi:10.1111/gcb.71056)
Supplement: Supplementary file 3 [file GCB-32-e71056-s004.docx]

**Supplementary Results**

**Effect of warming on FA levels**

Total FA concentrations showed increasing trends in primary producers under low and medium warming, with moderate support for an 11% increase under medium warming (Figure S6). SFA concentration increases were very strongly supported with rises of 13-17% across all warming levels. In contrast, high warming led to declines in PUFA concentrations of 18% with very strong support and declines in EPA and ALA omega-3 concentrations of 17-24% with strong to very strong support. Increases in DHA under low and medium warming, and a decrease under high warming were not statistically significant. Omega-6 FA concentrations showed increasing trends with warming, but these were not statistically supported (Figure S6).

In invertebrates, effect sizes were generally of larger magnitude with higher warming levels (Figure S6). Total FA concentrations declined by 26% under high warming with moderate support. Trends suggested increases in SFA concentrations, and decreases in PUFA and MUFA, although these shifts were not statistically supported. ALA reduced under high warming by 29%, and EPA under medium and high warming by 35-49% with moderate to very strong support. In contrast, DHA omega-3 concentrations tended to increase, with marginal support for a 55% rise under high warming (85% CIs slightly overlapped zero). Trends for omega-6 FAs were mixed but statistical support was only found for total omega-6 increasing by 15% under low warming with very strong support (Figure S6).

In fish, warming resulted in consistent declines in FA concentrations, with larger reductions under high warming (Figure S6). Total FA concentrations and DHA concentrations decreased by 43% and 50%, respectively, under high warming with very strong support. Across warming levels, SFA, PUFA, MUFA, total omega-3 and EPA concentrations declined by 15-27% with moderate to very strong support. ALA also decreased but this was only marginally supported under low warming, and no data was available for high warming levels. Omega-6 FAs also declined, with total omega-6, LA, and AA concentrations decreasing by 14-54% under low and high warming levels with moderate to very strong support (Figure S6).

*Model heterogeneity*

Residual heterogeneity was significant (QE = 30-51770, p < 0.01) across all models for proportions and concentrations, indicating that additional sources of variation in effect sizes remain beyond that explained by moderators and expected from sampling error (Nakagawa et al., 2023) (Table S7). The proportion of variance due to true heterogeneity was very high in all proportion models (I^2^ _total_ > 85%) and concentration models (I^2^ _total_ > 75%), except for ARA and total omega-3 concentrations in fish, where heterogeneity was still substantial (I^2^ _total_ = 68.6-74.0 %). This indicates that a large proportion of the residual variation reflects true heterogeneity across random effect levels (i.e., studies, taxa, experiments, and effect sizes) rather than sampling error (Nakagawa et al., 2023; Senior et al., 2016). The distribution of this true heterogeneity varied greatly across hierarchical random effect levels: between-study I^2^ = 0.0–98.6 %, between-taxa I^2^ = 0.0–63.6 %, between-experiments I^2^ = 0.0–87.8 %, between-effect size I^2^ = 0.0–93.3 %. This showed that sources of variability operate at multiple levels and differ substantially among models. Overall, models explained ≥ 50% of the variation in *lnRR*, except for ARA proportions in primary producers (R^2^_c_ = 0.38), EPA and total omega-3 proportions, PUFA, total omega-3, total omega-6 concentrations, and the omega-3: omega-6 ratio in invertebrates (R^2^_c_ = 0.16–0.47), MUFA proportions and concentrations, SFA concentrations, and total FA concentrations in fish (R^2^_c_ = 0.20–0.48) (Table S7).

**Effect of acidification on FA levels**

Overall trends in primary producers suggest FA concentrations tend to decrease more as acidification increases, with low acidification often associated with increases in FAs and high acidification with decreases (Figure S7). Under high acidification, total FA and DHA concentrations decreased by 15-24% with moderate support. Under low acidification, MUFA and SFA concentrations increased by 12-34% with moderate to very strong support. Under low and medium acidification, EPA omega-3 and LA omega-6 increased by 11-37% with strong to very strong support, and AA omega-6 increased under medium acidification by 15% with moderate support. In invertebrates, there were very few statistically supported results. PUFA concentrations increased by 69% and EPA by 34% under high acidification with very strong support. In fish, data availability was sparse with significant changes only seen in PUFA and DHA concentrations which decreased by 19-22% under medium acidification with moderate support (Figure S7).

*Model heterogeneity*

Residual heterogeneity was significant in 47/62 models (QE = 17-18740, p < 0.05) for proportions and concentrations, indicating that for 15 models, remaining variance after accounting for moderators was likely due to sampling error (Table S8). The proportion of residual variance due to true heterogeneity and not attributable to sampling error was very high (I^2^ _total_ > 80%) in 45/62 models, substantial (50% < I^2^ _total_ < 80%) in 3/62 models, moderate (25% < I^2^ _total_ < 50%) in 7/62 models, and low (I^2^ _total_ < 25%) in 7/62 models. The distribution of true heterogeneity varied greatly across hierarchical random effect levels: between-study I^2^ = 0.0-79.7 %, between-taxa I^2^ = 0.0-99.0 %, between-experiments I^2^ = 0.0 – 99.0 %, between-effect size I^2^ = 0.0-98.9 %. 50/62 models explained ≥ 50% of variation in *lnRR* (R^2^_c_), 6/62 models explained between 25% and 50% of variation, whilst 6/62 models explained < 25% (Table S8).

**Influence of moderators on FA responses to warming**

In primary producers, warming effects on FA concentrations varied significantly across climate zone origin, functional group, habitat type, control temperature, exposure duration, and life stage (Figure S8). Polar species had larger decreases in total FA (-38% ± 19.4), ALA (-49% ± 20.5), EPA (-51% ± 25.2), and AA (-90% ± 128.7) than temperate (total FA: 8% ± 7.5; ALA: 2% ± 12.4; EPA: 1% ± 12.1; AA: 44% ± 30.5), tropical (total FA: 51% ± 20.8), and cosmopolitan (ALA: 8% ± 19.4) species. Total omega-6 concentrations increased more in cosmopolitan species (34% ± 10.1) than temperate species (-7% ± 8.1). Macro primary producers had larger decreases in DHA (-62% ± 50.7) and total omega-3 (-17% ± 5.8) than diatoms (total omega-3: 37% ± 19.3) and other microalgae (DHA: 32% ± 17.0; total omega-3: 34% ± 15.1). Total omega-3 increased in pelagic species (29% ± 12.9) but decreased in benthic (-17% ± 5.2), and PUFA decreased more in species from other habitat zones (-43% ± 21.5) than in pelagic species (0% ± 6.3). Life stage 3 (adult or stationary growth phase) had larger declines in ALA (-22% ± 13.5), DHA (-23% ± 19.3), total omega-3 (-19% ± 13.4), and PUFA (-21% ± 11.1) than life stage 2 (immature or exponential growth phase; ALA: 13% ± 13.0; DHA: 29% ± 17.1; total omega-3: 46% ± 16.3; PUFA: 8% ± 11.2). Long exposure (>336 hrs) led to the largest increases in AA (111% ± 34.0), MUFA (44% ± 9.9), and SFA (53% ± 10.0) than intermediate (228-336 hrs) (AA: -12% ± 20.0; MUFA: -5% ± 7.4; SFA: 4% ± 7.9) and short exposures (≤228 hrs) (AA: 5% ± 28.5; MUFA: -13 ± 7.8; SFA: 7% ± 7.4). Intermediate exposure led to larger declines across EPA (-30% ± 12.7) and PUFA (-20% ± 7.2) than short (EPA: -7% ± 11.6) or long (PUFA: 7% ± 9.6) exposure. LA concentrations increased more as the control temperature increased (β = 53% ± 21.9) (Figure S8).

In invertebrates, body size, exposure duration, and life stage significantly influenced the effect of temperature on FA concentrations (Figure S8). Moderate exposure duration led larger declines in total omega-3 (-22% ± 6.9) than long exposure (>720 hrs) (16% ± 14.7). DHA concentrations increased much more in the 2-20 mm body size class (193% ± 32.3) than 0.2-2 mm (-20% ± 26.6) and >200 mm (-76% ± 84.8) body size classes, and in larvae (207% ± 57.8) than juveniles (25% ± 74.4) and adults (-20% ± 29.5) (Figure S8).

In fish, body size, longevity, trophic level, exposure duration, and tissue type moderated the effects of temperature on FA concentrations (Figure S8). Intermediate body sizes (35-103 cm) also had larger declines in total omega-3 (-53% ± 11.1), total omega-6 (-71% ± 26.7), and PUFA (-58% ± 17.5) than small (≤35 cm) (total omega-3: -23% ± 3.6; total omega-6: -22% ± 10.0; PUFA: -22% ± 9.3) and large (> 103 cm) (total omega-3: -28% ± 6.5; total omega-6: -26% ± 8.8; PUFA: -27% ± 18.2) body sizes. Intermediate exposure durations (240-2920.8 hrs) led to larger declines in total omega-3 (-53% ± 11.8), LA (-47% ± 23.2), total omega-6 (-71% ± 26.7), and PUFA (-46% ± 15.7) than short (≤240 hrs) (total omega-3: -25% ± 4.4; total omega-6: -24% ± 7.5; PUFA: -23% ± 9.3) or long (>2920.8 hrs) (total omega-3: -26% ± 11.7; LA: 13% ± 15.7; total omega-6: -25% ± 14.3) exposures. DHA decreased marginally (p = 0.054) more in liver tissue (-30% ± 15.7) than muscle tissue (25% ± 20.2). ALA and total omega-6 decreased more with greater longevity (ALA: β = -33% ± 12.4; omega-6: β = -37% ± 16.9), and SFA concentrations increased more with higher trophic levels (β = 12% ± 4.4) (Figure S8).

*Model Heterogeneity*

Residual heterogeneity was significant in 627/643 models (QE = 19-49295, p < 0.05) and in 83/90 models where the moderator was significant (QE = 0-37056, p < 0.05), indicating that the remaining variance after accounting for moderators was likely due to sampling error in 16 models overall and 7 models in the significant moderator subset, (Table S9). The proportion of residual variance due to true heterogeneity and not attributable to sampling error was very high (I^2^ _total_ > 80%) in 593/643 models (79/90 significant-moderator models), substantial (50% < I^2^ _total_ < 80% ) in 41/643 models (0/90), moderate (25% < I^2^ _total_ < 50% ) in 3/643 models (3/90), and low (I^2^ _total_ < 25%) in 6/643 models (3/90). The distribution of true heterogeneity varied greatly across hierarchical random effect levels: between-study I^2^ = 0.0-99.1 % (0.0-99.4 % for significant moderator models), between-taxa I^2^ = 0.0-93.8 % (0.0-70.3 %), between-experiments I^2^ = 0.0-92.4 % (0.0-88.2 %), between-effect size I^2^ = 0.0-98.6 % (0.0-98.5 %). 196/643 models (31/90 significant moderator models) explained ≥ 80% of variation in *lnRR* (R^2^_c_), 357/643 models (50/90) explained between 50% and 80% of variation, 71/643 models (9/90) explained between 25% and 50% of variation, whilst 19/643 models (0/90) explained < 25% (Table S9).
