# Supplementary material for: The Effects of Ocean Warming and Acidification on Fatty Acid Contents of Marine Organisms: A Global Meta‐Analysis
Source: Glob Chang Biol. 2026 Aug 19;32(8):e71056. doi: 10.1111/gcb.71056 (PMC13487444; doi:10.1111/gcb.71056)
Supplement: Supplementary file 4 [file GCB-32-e71056-s005.docx]

**Supplementary Figures**

**Figure S1.** PRISMA flow diagram depicting the number of studies identified in the literature search and then included or excluded during each stage of screening. The total number of studies is represented by n, whilst values in parentheses following n indicate concentration (Conc) and proportion (Prop) counts, respectively.

**Figure S2.** Histograms showing the distribution of (a) maximum body size across fish; and (b) the duration across primary producers, invertebrates, and fish. Dashed vertical lines indicate the subcategory boundaries as defined based on the data distribution.

**Figure S3.** Funnel plots of effect sizes for (a) warming and (b) acidification, plotted against precision (inverse of SE) for each FA group across primary producers, invertebrates, and fish. Dashed vertical lines indicate the mean effect size.

**Figure S4.** Histograms for (a) warming and (b) acidification showing the distribution of log response ratio (*lnRR*) values for each FA group across primary producers, invertebrates, and fish.

**Figure S5.** Histograms showing the distribution of (a) warming and (b) acidification increase values across primary producers, invertebrates, and fish. Dashed vertical lines indicate the subcategory boundaries as defined based on the data distribution, delineating low, medium and high levels respectively.

**Figure S6.** Percentage changes in FA absolute concentration response to three warming levels (≤6°C, 6-10°C, and >10°C) calculated from average log response ratio (*lnRR*) slope coefficient estimates (*β*). The thick, medium, and narrow horizontal bars denote 85%, 90%, and 95% confidence intervals, respectively, and the dashed vertical line represents a null effect. Filled circles are where 85% confidence intervals do not overlap zero. Estimates to the right of zero indicate increases under warming, whilst those to the left indicate decreases. Results were considered to have very strong, strong, and moderate support at the 95%, 90%, and 85% confidence levels, respectively. Open circles show effects whose 85% confidence intervals overlap zero, indicating no consistent directional effect. Species represented across all models: primary producers (n=45), invertebrates (n=15), fish (n=9). FA acronyms as per Figure 1.

**Figure S7.** Percentage changes in FA absolute concentrations in response to three acidification levels (≤582 µatm, 582-1700 µatm, and >1700 µatm) calculated from average log response ratio (*lnRR*) slope coefficient estimates (*β*). The thick, medium, and narrow horizontal bars denote 85%, 90%, and 95% confidence intervals, respectively, and the dashed vertical line represents a null effect. Estimates to the right of zero indicate increases under warming, whilst those to the left indicate decreases. Results were considered to have very strong, strong, and moderate support at the 95%, 90%, and 85% confidence levels, respectively. Open circles show effects whose 85% confidence intervals overlap zero, indicating no consistent directional effect. Species represented across all models: primary producers (n=15), invertebrates (n=4), fish (n=3). FA acronyms as per Figure 1.

**Figure S8.** Heat maps showing the effects of warming on FA concentrations for (a) primary producers, (b) invertebrates, and (c) fish. Rows represent FAs and columns represent moderators, with categorical moderators on the left, and continuous moderators on the right. Only significant results are shown (p<0.05; ANOVA for categorical moderators, slope [*β*] for continuous moderators), with grey cells indicating non-significant effects, and black cells indicating missing data. For categorical moderators, cells show the estimated FA warming effect (*lnRR*) for each subcategory: orange indicates an increase, blue a decrease, and white no change. Letters denote pairwise comparisons, with different letters indicating significant differences (p<0.05). For continuous moderators, cells show the slope (*β*) of the relationship between the FA warming effect (*lnRR*) and the moderator: orange indicates positive relationships and blue negative. FA acronyms as per Figure 1.
