# Supplementary material for: The Effects of Ocean Warming and Acidification on Fatty Acid Contents of Marine Organisms: A Global Meta‐Analysis
Source: Glob Chang Biol. 2026 Aug 19;32(8):e71056. doi: 10.1111/gcb.71056 (PMC13487444; doi:10.1111/gcb.71056)
Supplement: Supplementary file 5 [file GCB-32-e71056-s003.zip › Tables_SuppInfo.docx]

**Supplementary Tables**

**Table S1.** Variables assessed as moderators of warming effects on FA levels with their subcategories for three taxa groups: primary producers, invertebrates, and fish. Exclusion justifications and notes are indicated by superscripts: (1) insufficient data; (2) not informative; (3) highly unbalanced subcategories; (4) included, but with a largely reduced dataset.

| Moderator | Primary producers | Invertebrates | Fish | Categorical or numeric |
| --- | --- | --- | --- | --- |
| *Biological traits* | | | |  |
| Body size | No ^1^ | (mm)  ≤2  2-20  20-200  >200 | (cm)  ≤35  35-103  >103 | Categorical |
| Climate zone | Polar  Temperate  Tropical  Cosmopolitan | Polar  Temperate  Tropical  Cosmopolitan | Polar  Temperate  Tropical  Cosmopolitan | Categorical |
| Diet group | No ^2^ | Herbivore  Carnivore  Omnivore | No ^3^ | Categorical |
| Feeding type | No ^2^ | No ^1^ | Plankton-feeder  Macrofauna-predator  Variable | Categorical |
| Functional group | Diatom  Other microalgae  Macro primary producer | n/a | n/a | Categorical |
| Habitat | Benthic  Pelagic  Other | Benthic  Pelagic | No ^3^ | Categorical |
| Life stage | Stage 1 (larval or lag phase)  Stage 2 (immature or log phase)  Stage 3 (adult or stationary phase)  Stage 4 (decay phase) | Larval  Juvenile  Adult | Larval  Juvenile  Adult | Categorical |
| Longevity | No ^1^ | No ^1^ | Yes | Numeric |
| Mean preferred temperature | No ^1^ | Yes ^4^ | Yes | Numeric |
| Preferred temperature range | No ^1^ | Yes ^4^ (Prop.); No ^1^ (Conc.) | Yes | Numeric |
| Resilience to fishing pressure | No ^1^ | No ^1^ | Low  Medium  High ^4^ | Categorical |
| Trophic level | n/a | No ^1^ | yes | Numeric |
| Vulnerability to fishing | No ^1^ | No ^1^ | Yes | Numeric |
| Vulnerability to climate change | No ^1^ | No ^1^ | Yes ^4^ | Numeric |
| *Methodological* |  |  |  |  |
| Control temperature | Yes | Yes | Yes | Numeric |
| Experiment duration (hrs) | ≤228  228-336  >336 | ≤168  168-720  >720 | ≤240  240-2920.8  >2920.8 | Categorical |
| Tissue type | No ^2^ | No ^2^ | Whole body  Muscle  Liver  Other | Categorical |

**Table S2.** Results of Egger’s test examining the relationship between effect sizes and effective sample sizes for temperature. NA values indicate cases where Egger’s test could not be conducted due to insufficient variation or number of effective sample sizes.

|  |  | Warming | | Acidification | |
| --- | --- | --- | --- | --- | --- |
| FA group | **Taxa group** | **Prop.**  **p-value** | **Conc.**  **p-value** | **Prop.**  **p-value** | **Conc.**  **p-value** |
| ALA | primary producers | 0.35 | 0.72 | 0.89 | 0.58 |
|  | invertebrates | 0.76 | 0.07 | 0.08 | 0.27 |
|  | fish | 0.92 | 0.26 | 0.61 | 0.36 |
| EPA | primary producers | 0.43 | 0.50 | 0.82 | 0.69 |
|  | invertebrates | 0.74 | 0.16 | 0.83 | 0.70 |
|  | fish | 0.78 | 0.43 | 0.05 | 0.39 |
| DHA | primary producers | 0.92 | 0.72 | 0.44 | 0.87 |
|  | invertebrates | 0.95 | 0.46 | **0.03** | 0.82 |
|  | fish | 0.89 | 0.96 | 0.56 | 0.24 |
| Σω3 | primary producers | 0.54 | 0.60 | 0.84 | 0.95 |
|  | invertebrates | 0.69 | 0.52 | 0.53 | 0.14 |
|  | fish | 0.64 | **0.02** | 0.85 | NA |
| LA | primary producers | 0.42 | 0.64 | 0.41 | 0.30 |
|  | invertebrates | 0.18 | 0.07 | **0.02** | 0.08 |
|  | fish | 0.90 | 0.70 | 0.82 | NA |
| AA | primary producers | 0.67 | 0.80 | 0.82 | 0.64 |
|  | invertebrates | 0.79 | 0.08 | 0.07 | 0.63 |
|  | fish | 0.72 | 0.93 | 0.61 | 0.06 |
| Σω6 | primary producers | 0.60 | 0.85 | 0.38 | 0.98 |
|  | invertebrates | 0.43 | 0.26 | **0.03** | 0.45 |
|  | fish | 0.07 | 0.10 | 0.60 | NA |
| PUFA | primary producers | 0.80 | 0.61 | 0.58 | 0.41 |
|  | invertebrates | 0.47 | 0.13 | 0.79 | 0.26 |
|  | fish | 0.70 | 0.16 | 0.10 | NA |
| MUFA | primary producers | 0.94 | 0.55 | 0.69 | 0.18 |
|  | invertebrates | 0.71 | 0.11 | 0.68 | 0.09 |
|  | fish | 0.77 | 0.80 | 0.13 | NA |
| SFA | primary producers | 0.65 | 0.80 | 0.66 | 0.57 |
|  | invertebrates | 0.29 | 0.28 | 0.24 | 0.11 |
|  | fish | 0.17 | 0.78 | 0.32 | NA |
| ω3 : ω6 | primary producers | NA |  | NA |  |
|  | invertebrates | 0.60 |  | NA |  |
|  | fish | 0.81 |  | NA |  |
| Total FA | primary producers |  | 0.82 |  | 0.83 |
|  | invertebrates |  | 0.26 |  | 0.19 |
|  | fish |  | 0.11 |  | NA |

**Table S3.** Species included across analyses. OW = ocean warming, OA = ocean acidification. Numbers in square brackets indicate how many studies included a specimen belonging to the specified genus that was not identified to the species level.

| Primary producers | | | |
| --- | --- | --- | --- |
| Species | **Class** | **Phylum** | **Variable** |
| *Achnanthes brevipes* | Bacillariophyceae | Bacillariophyta | OW |
| *Akashiwo sanguinea* | Dinophyceae | Myzozoa | OA |
| *Amphidinium massartii* | Dinophyceae | Myzozoa | OW |
| *Amphora arenaria* | Bacillariophyceae | Bacillariophyta | OW |
| *Amphora* sp. [1] | Bacillariophyceae | Bacillariophyta | OW |
| *Ascophyllum nodosum* | Phaeophyceae | Ochrophyta | OW |
| *Bryopsis hypnoides* | Ulvophyceae | Chlorophyta | OW |
| *Chaetoceros brevis* | Bacillariophyceae | Bacillariophyta | OW |
| *Chaetoceros calcitrans* | Bacillariophyceae | Bacillariophyta | OW |
| *Chaetoceros muelleri* | Bacillariophyceae | Bacillariophyta | OA & OW |
| *Chaetoceros neogracile* | Bacillariophyceae | Bacillariophyta | OW |
| *Chaetoceros* sp. [3] | Bacillariophyceae | Bacillariophyta | OW |
| *Chaetoceros tenuissimus* | Bacillariophyceae | Bacillariophyta | OW |
| *Chaetoceros wighamii* | Bacillariophyceae | Bacillariophyta | OW |
| *Chlamydomonas reinhardtii* | Chlorophyceae | Chlorophyta | OA |
| *Chlamydomonas* sp. [4] | Chlorophyceae | Chlorophyta | OW |
| *Chlorella* sp. [4] | Trebouxiophyceae | Chlorophyta | OW |
| *Chlorella stigmatophora* | Trebouxiophyceae | Chlorophyta | OW |
| *Chlorella vulgaris* | Trebouxiophyceae | Chlorophyta | OA & OW |
| *Chromera velia* | Colpodellophyceae | Chromerida | OW |
| *Cladocopium* sp. [2] | Dinophyceae | Myzozoa | OW |
| *Cocconeis peltoides* | Bacillariophyceae | Bacillariophyta | OW |
| *Codium papillatum* | Ulvophyceae | Chlorophyta | OW |
| *Colpomenia sinuosa* | Phaeophyceae | Ochrophyta | OW |
| *Crypthecodinium cohnii* | Dinophyceae | Myzozoa | OW |
| *Cryptomonas* sp. [1] | Cryptophyceae | Cryptophyta | OW |
| *Cylindrotheca closterium* | Bacillariophyceae | Bacillariophyta | OW |
| *Cylindrotheca fusiformis* | Bacillariophyceae | Bacillariophyta | OA & OW |
| *Cymodocea nodosa* | Magnoliopsida | Tracheophyta | OW |
| *Derbesia tenuissima* | Ulvophyceae | Chlorophyta | OW |
| *Diacronema lutheri* | Pavlovophyceae | Haptophyta | OW |
| *Dixioniella grisea* | Rhodellophyceae | Chlorophyta | OW |
| *Dunaliella salina* | Chlorophyceae | Chlorophyta | OA |
| *Dunaliella* sp. [1] | Chlorophyceae | Chlorophyta | OW |
| *Effrenium voratum* | Dinophyceae | Myzozoa | OW |
| *Feldmannia mitchelliae* | Phaeophyceae | Ochrophyta | OW |
| *Fucus serratus* | Phaeophyceae | Ochrophyta | OW |
| *Gephyrocapsa huxleyi* | Coccolithophyceae | Haptophyta | OA & OW |
| *Gymnodinium* sp. [1] | Dinophyceae | Myzozoa | OA |
| *Heterocapsa* sp. [1] | Dinophyceae | Myzozoa | OA |
| *Isochrysis galbana* | Coccolithophyceae | Haptophyta | OW |
| *Isochrysis* sp. [3] | Coccolithophyceae | Haptophyta | OW |
| *Iyengaria stellata* | Phaeophyceae | Ochrophyta | OW |
| *Kryptoperidinium triquetrum* | Dinophyceae | Myzozoa | OW |
| *Laminaria digitata* | Phaeophyceae | Ochrophyta | OW |
| *Leptocylindrus danicus* | Bacillariophyceae | Bacillariophyta | OW |
| *Microchloropsis salina* | Eustigmatophyceae | Ochrophyta | OW |
| *Mychonastes homosphaera* | Chlorophyceae | Chlorophyta | OW |
| *Nannochloropsis oceanica* | Eustigmatophyceae | Ochrophyta | OA & OW |
| *Nannochloropsis oculata* | Eustigmatophyceae | Ochrophyta | OW |
| *Nannochloropsis* sp. [3] | Eustigmatophyceae | Ochrophyta | OW |
| *Nanozostera noltei* | Magnoliopsida | Tracheophyta | OW |
| *Navicula digitoradiata* | Bacillariophyceae | Bacillariophyta | OW |
| *Navicula gregaria* | Bacillariophyceae | Bacillariophyta | OW |
| *Navicula* sp. [2] | Bacillariophyceae | Bacillariophyta | OW |
| *Nitzschia closterium* | Bacillariophyceae | Bacillariophyta | OW |
| *Nitzschia lecointei* | Bacillariophyceae | Bacillariophyta | OA & OW |
| *Nitzschia paleacea* | Bacillariophyceae | Bacillariophyta | OW |
| *Nitzschia* sp. [1] | Bacillariophyceae | Bacillariophyta | OW |
| *Odontella aurita* | Bacillariophyceae | Bacillariophyta | OW |
| *Ostreococcus tauri* | Mamiellophyceae | Chlorophyta | OW |
| *Palmaria decipiens* | Florideophyceae | Rhodophyta | OW |
| *Palmaria palmata* | Florideophyceae | Rhodophyta | OW |
| *Phaeocystis antarctica* | Pyramimonadophyceae | Chlorophyta | OA |
| *Phaeodactylum tricornutum* | Bacillariophyceae | Bacillariophyta | OA & OW |
| *Phyllospora comosa* | Phaeophyceae | Ochrophyta | OW |
| *Picochlorum atomus* | Trebouxiophyceae | Chlorophyta | OW |
| *Picochlorum maculatum* | Trebouxiophyceae | Chlorophyta | OW |
| *Pinguiococcus pyrenoidosus* | Pinguiophyceae | Ochrophyta | OW |
| *Polysiphonia coacta* | Florideophyceae | Rhodophyta | OW |
| *Porphyridium purpureum* | Porphyridiophyceae | Rhodophyta | OW |
| *Posidonia oceanica* | Magnoliopsida | Tracheophyta | OW |
| *Pseudopleurochloris antarctica* | Xanthophyceae | Ochrophyta | OW |
| *Pterocladia heteroplatos* | Florideophyceae | Rhodophyta | OW |
| *Pyramimonas gelidicola* | Pyramimonadophyceae | Chlorophyta | OA |
| *Rhodella violacea* | Rhodellophyceae | Rhodophyta | OW |
| *Rhodomonas salina* | Cryptophyceae | Cryptophyta | OA & OW |
| *Rhodomonas* sp. [3] | Cryptophyceae | Cryptophyta | OA & OW |
| *Saccharina japonica* | Phaeophyceae | Ochrophyta | OA |
| *Sargassum boveanum* | Phaeophyceae | Ochrophyta | OW |
| *Skeletonema marinoi* | Bacillariophyceae | Bacillariophyta | OW |
| *Skeletonema pseudocostatum* | Bacillariophyceae | Bacillariophyta | OA |
| *Symbiodinium microadriaticum* | Dinophyceae | Myzozoa | OW |
| *Synedra* sp. [1] | Bacillariophyceae | Bacillariophyta | OW |
| *Tetraselmis subcordiformi* | Chlorodendrophyceae | Chlorophyta | OW |
| *Tetraselmis suecica* | Chlorodendrophyceae | Chlorophyta | OW |
| *Tetraselmis tetrathele* | Chlorodendrophyceae | Chlorophyta | OW |
| *Thalassiosira baltica* | Bacillariophyceae | Bacillariophyta | OW |
| *Thalassiosira pseudonana* | Bacillariophyceae | Bacillariophyta | OA & OW |
| *Thalassiosira* sp. [1] | Bacillariophyceae | Bacillariophyta | OW |
| *Thalassiosira weissflogii* | Bacillariophyceae | Bacillariophyta | OA |
| *Ulva compressa* | Ulvophyceae | Chlorophyta | OA |
| *Ulva fenestrata* | Ulvophyceae | Chlorophyta | OA & OW |
| *Ulva intestinalis* | Ulvophyceae | Chlorophyta | OW |
| *Ulva lactuca* | Ulvophyceae | Chlorophyta | OA & OW |
| *Ulva rigida* | Ulvophyceae | Chlorophyta | OW |
| *Zostera marina* | Magnoliopsida | Tracheophyta | OW |
|  |  | Haptophyta | OW |
| Invertebrates | | | |
| Species | **Class** | **Phylum** | **Variable** |
| *Acartia bifilosa* | Copepoda | Arthropoda | OA |
| *Acartia hudsonica* | Copepoda | Arthropoda | OA & OW |
| *Acartia tonsa* | Copepoda | Arthropoda | OA & OW |
| *Acropora intermedia* | Anthozoa | Cnidaria | OW |
| *Amonardia* sp. [1] | Copepoda | Arthropoda | OW |
| *Artemia franciscana* | Branchiopoda | Arthropoda | OW |
| *Artemia sinica* | Branchiopoda | Arthropoda | OA |
| *Aurelia aurita* | Scyphozoa | Cnidaria | OW |
| *Brachionus koreanus* | Eurotatoria | Rotifera | OW |
| *Brachionus plicatilis* | Eurotatoria | Rotifera | OW |
| *Brachionus rotundiformis* | Eurotatoria | Rotifera | OW |
| *Calanus finmarchicus* | Copepoda | Arthropoda | OW |
| *Crassadoma gigantea* | Gastropoda | Mollusca | OA & OW |
| *Dicathais orbita* | Gastropoda | Mollusca | OA & OW |
| *Euphausia superba* | Malacostraca | Arthropoda | OA |
| *Eurytemora affinis.affinis* | Copepoda | Arthropoda | OA |
| *Exaiptasia diaphana* | Anthozoa | Cnidaria | OW |
| *Hediste diversicolor* | Polychaeta | Annelida | OW |
| *Lepeophtheirus salmonis.salmonis* | Copepoda | Arthropoda | OA |
| *Magallana gigas* | Bivalvia | Mollusca | OA |
| *Montipora digitata* | Anthozoa | Cnidaria | OW |
| *Mytilus chilensis* | Bivalvia | Mollusca | OA |
| *Octopus maya* | Cephalapoda | Mollusca | OW |
| *Palaemon elegans* | Malacostraca | Arthropoda | OA & OW |
| *Paracalanus* sp. [2] | Copepoda | Arthropoda | OA & OW |
| *Paracyclopina nana* | Copepoda | Arthropoda | OW |
| *Paralithodes camtschaticus* | Malacostraca | Arthropoda | OW |
| *Paralithodes platypus* | Malacostraca | Arthropoda | OW |
| *Platychelipus littoralis* | Copepoda | Arthropoda | OW |
| *Portunus armatus* | Malacostraca | Arthropoda | OW |
| *Saccostrea glomerata* | Bivalvia | Mollusca | OW |
| *Sclerophytum capitale* | Anthozoa | Cnidaria | OW |
| *Scylla olivacea* | Malacostraca | Arthropoda | OW |
| *Scylla serrata* | Malacostraca | Arthropoda | OW |
| *Tisbe* sp. [1] | Copepoda | Arthropoda | OW |
| *Turbo militaris* | Gastropoda | Mollusca | OA & OW |
| Fish | | | |
| Species | **Class** | **Phylum** | **Variable** |
| *Acanthopagrus australis* | Teleostei | Chordata | OA & OW |
| *Amphiprion ocellaris* | Teleostei | Chordata | OW |
| *Argyrosomus regius* | Teleostei | Chordata | OW |
| *Dicentrarchus labrax* | Teleostei | Chordata | OW |
| *Fundulus heteroclitus* | Teleostei | Chordata | OW |
| *Gadus chalcogrammus* | Teleostei | Chordata | OA |
| *Gadus macrocephalus* | Teleostei | Chordata | OA |
| *Inimicus japonicus* | Teleostei | Chordata | OW |
| *Lepidonotothen squamifrons* | Teleostei | Chordata | OW |
| *Mugil cephalus* | Teleostei | Chordata | OW |
| *Notothenia rossii* | Teleostei | Chordata | OA & OW |
| *Pachycara brachycephalum* | Teleostei | Chordata | OW |
| *Paralabrax humeralis* | Teleostei | Chordata | OW |
| *Salmo salar* | Teleostei | Chordata | OW |
| *Sciaenops ocellatus* | Teleostei | Chordata | OA |
| *Sparus aurata* | Teleostei | Chordata | OW |
| *Trematomus bernacchii* | Teleostei | Chordata | OW |
| *Zoarces americanus* | Teleostei | Chordata | OW |
| *Zoarces viviparus* | Teleostei | Chordata | OW |

**Table S4.** The number of effect sizes (k), with the number of papers and experiments in parenthesis, included in each warming model for primary producers, invertebrates, and fish.

|  | Primary producers  *k* (papers; experiments) | Invertebrates  *k* (papers; experiments) | Fish  *k* (papers; experiments) |
| --- | --- | --- | --- |
| FA Proportion | | | |
| ALA | 243 (30, 107) | 78 (21, 47) | 77 (15, 50) |
| EPA | 451 (60, 200) | 89 (24, 56) | 105 (22, 67) |
| DHA | 275 (40, 126) | 87 (23, 54) | 104 (22, 66) |
| Σω3 | 101 (11, 51) | 39 (16, 26) | 69 (13, 40) |
| LA | 357 (47, 154) | 51 (14, 32) | 87 (17, 57) |
| AA | 225 (37, 116) | 81 (21, 48) | 83 (19, 47) |
| Σω6 | 48 (7, 35) | 36 (15, 25) | 69 (13, 40) |
| PUFA | 424 (49, 194) | 84 (22, 53) | 96 (18, 59) |
| MUFA | 406 (48, 193) | 84 (22, 52) | 96 (18, 59) |
| SFA | 407 (48, 193) | 84 (22, 52) | 96 (18, 59) |
| ω3 : ω6 | 98 (11, 52) | 39 (16, 26) | 91 (17, 60) |
| FA Concentration | | | |
| ALA | 181 (22, 76) | 30 (9, 18) | 19 (5, 13) |
| EPA | 308 (33, 117) | 45 (13, 29) | 35 (10, 21) |
| DHA | 195 (23, 72) | 44 (12, 28) | 35 (10, 21) |
| Σω3 | 82 (7, 42) | 16 (7, 11) | 16 (5, 13) |
| LA | 86 (16, 42) | 34 (9, 20) | 25 (6, 14) |
| AA | 132 (18, 53) | 39 (11, 23) | 35 (10, 21) |
| Σω6 | 40 (4, 28) | 16 (7, 11) | 16 (5, 13) |
| PUFA | 293 (24, 118) | 41 (11, 26) | 17 (5, 9) |
| MUFA | 280 (24, 118) | 43 (12, 27) | 22 (6, 14) |
| SFA | 280 (24, 118) | 46 (13, 28) | 22 (6, 14) |
| Total FA | 314 (31, 133) | 44 (13, 28) | 20 (6, 12) |

**Table S5.** The number of effect sizes (k), with the number of papers and experiments in parenthesis, included in each acidification model for primary producers, invertebrates, and fish.

|  | Primary producers  *k* (papers; experiments) | Invertebrates  *k* (papers; experiments) | Fish  *k* (papers; experiments) |
| --- | --- | --- | --- |
| FA Proportion | | | |
| ALA | 33 (10, 29) | 47 (10, 19) | - |
| EPA | 50 (11, 40) | 58 (12, 22) | 8 (4, 5) |
| DHA | 39 (10, 29) | 59 (13, 23) | 8 (4, 5) |
| Σω3 | 12 (2, 5) | 14 (6, 12) | - |
| LA | 20 (6, 16) | 42 (8, 15) | 8 (4, 5) |
| AA | 11 (5, 7) | 40 (9, 17) | 8 (4, 5) |
| Σω6 | 12 (2, 5) | 14 (6, 12) | - |
| PUFA | 66 (14, 48) | 45 (12, 23) | 9 (4, 6) |
| MUFA | 65 (15, 49) | 43 (12, 21) | 9 (4, 6) |
| SFA | 68 (15, 50) | 43 (12, 21) | 9 (4, 6) |
| ω3 : ω6 | 15 (3, 8) | 14 (6, 12) | - |
| FA Concentration | | | |
| ALA | 31 (8, 27) | 7 (3, 5) | - |
| EPA | 43 (8, 35) | 18 (5, 8) | 7 (3, 4) |
| DHA | 33 (7, 25) | 19 (6, 9) | 7 (3, 4) |
| Σω3 | 12 (2, 5) | 4 (1, 2) | - |
| LA | 20 (7, 16) | 8 (4, 6) | - |
| AA | 7 (3, 4) | 8 (4, 6) | 7 (3, 4) |
| Σω6 | 12 (2, 5) | 4 (1, 2) | - |
| PUFA | 49 (11, 41) | 18 (5, 9) | 6 (2, 3) |
| MUFA | 51 (12, 43) | 18 (5, 9) | 6 (2, 3) |
| SFA | 51 (12, 43) | 18 (5, 9) | 6 (2, 3) |
| Total FA | 51 (12, 43) | 18 (5, 9) | 6 (2, 3) |

**Table S6.** The number of effect sizes (k) within each subcategory included in each model for primary producers, invertebrates, and fish.

| Proportions | | | | | | | | | | | | |
| --- | --- | --- | --- | --- | --- | --- | --- | --- | --- | --- | --- | --- |
| Primary producers | | | | | | | | | | | | |
| Moderator | **Subcategory** | **ALA** | **EPA** | **DHA** | **Σω3** | **LA** | **AA** | **Σω6** | **PUFA** | **MUFA** | **SFA** | **ω3:ω6** |
| Climate | cosmopolitan | 33 | 36 | 34 | 12 | 22 | 15 | 12 | 47 | 49 | 49 | 24 |
|  | polar | 13 | 15 | 5 | NA | 21 | NA | NA | 24 | 23 | 24 | NA |
|  | temperate | 109 | 249 | 134 | 78 | 184 | 121 | 34 | 227 | 227 | 227 | 70 |
|  | tropical | 39 | 56 | 52 | 8 | 53 | 36 | NA | 45 | 45 | 45 | NA |
| Duration | >336 | 44 | 72 | 32 | 32 | 49 | 15 | NA | 70 | 70 | 70 | 36 |
|  | ≤228 | 61 | 139 | 69 | 26 | 108 | 78 | 16 | 107 | 88 | 88 | 17 |
|  | 228-336 | 55 | 110 | 59 | 36 | 111 | 90 | 28 | 121 | 122 | 123 | 28 |
| Functional group | diatom | 44 | 151 | 103 | 14 | 99 | 34 | 6 | 136 | 121 | 121 | 7 |
|  | macro | 47 | 74 | 6 | 72 | 79 | 42 | 40 | 80 | 82 | 82 | 76 |
|  | other microalgae | 152 | 226 | 166 | 15 | 179 | 149 | 3 | 208 | 203 | 204 | 15 |
| Life stage | stage1 | NA | 3 | NA | NA | 3 | 3 | NA | 3 | 3 | 3 | NA |
|  | stage2 | 117 | 222 | 167 | 26 | 166 | 114 | 7 | 197 | 193 | 193 | 8 |
|  | stage3 | 111 | 169 | 87 | 39 | 143 | 63 | 6 | 178 | 162 | 163 | 54 |
|  | stage4 | NA | 1 | 1 | NA | NA | NA | NA | 1 | 1 | 1 | NA |
| Habitat | benthic | 53 | 112 | 28 | 72 | 100 | 51 | 40 | 117 | 119 | 119 | 76 |
|  | other | 16 | 23 | 27 | NA | 10 | 11 | NA | 16 | 16 | 16 | NA |
|  | pelagic | 163 | 306 | 209 | 25 | 236 | 152 | 9 | 284 | 264 | 265 | 22 |
| Control temp | NA | 243 | 451 | 275 | 101 | 357 | 225 | 49 | 424 | 406 | 407 | 98 |
| Invertebrates | | | | | | | | | | | | |
| Moderator | **Subcategory** | **ALA** | **EPA** | **DHA** | **Σω3** | **LA** | **AA** | **Σω6** | **PUFA** | **MUFA** | **SFA** | **ω3:ω6** |
| Body size (mm) | >200 | 34 | 34 | 33 | 11 | 32 | 34 | 8 | 31 | 31 | 31 | 8 |
|  | 0.2-2 | 15 | 21 | 20 | 9 | 11 | 16 | 9 | 18 | 20 | 20 | 12 |
|  | 20-200 | 15 | 19 | 19 | 17 | 6 | 17 | 17 | 21 | 19 | 19 | 17 |
|  | 2-20 | 14 | 15 | 15 | 2 | 2 | 14 | 2 | 14 | 14 | 14 | 2 |
| Climate | cosmopolitan | 6 | 6 | 6 | NA | 6 | 6 | NA | 6 | 6 | 6 | NA |
|  | temperate | 47 | 54 | 52 | 28 | 38 | 46 | 25 | 51 | 49 | 49 | 28 |
|  | tropical | 21 | 25 | 25 | 11 | 3 | 25 | 11 | 27 | 29 | 29 | 11 |
| Duration | >720 | 23 | 23 | 23 | 15 | 21 | 21 | 12 | 21 | 19 | 19 | 12 |
|  | ≤168 | 18 | 22 | 22 | 4 | NA | 22 | 4 | 24 | 24 | 24 | 4 |
|  | 168-720 | 37 | 43 | 42 | 18 | 29 | 37 | 18 | 39 | 39 | 39 | 21 |
| Life stage | adult | 28 | 38 | 37 | 26 | 15 | 33 | 26 | 41 | 41 | 41 | 29 |
|  | juvenile | 34 | 35 | 34 | 13 | 32 | 32 | 10 | 31 | 31 | 31 | 10 |
|  | larvae | 12 | 12 | 12 | NA | NA | 12 | NA | 12 | 12 | 12 | NA |
| Diet group | carnivore | 25 | 25 | 24 | 10 | 23 | 25 | 10 | 22 | 20 | 20 | 10 |
|  | herbivore | 26 | 28 | 27 | 12 | 15 | 25 | 12 | 24 | 26 | 26 | 15 |
|  | omnivore | 27 | 36 | 36 | 17 | 13 | 31 | 14 | 36 | 36 | 36 | 14 |
| Habitat | benthic | 46 | 51 | 50 | 34 | 36 | 49 | 31 | 45 | 43 | 43 | 31 |
|  | pelagic | 32 | 38 | 37 | 5 | 15 | 32 | 5 | 39 | 41 | 41 | 8 |
| Control temp | NA | 78 | 89 | 87 | 39 | 51 | 81 | 36 | 84 | 84 | 84 | 39 |
| Mean temp | NA | 33 | 38 | 37 | 12 | 33 | 35 | 9 | 37 | 35 | 35 | 9 |
| Temp range | NA | 33 | 38 | 37 | 12 | 33 | 35 | 9 | 37 | 35 | 35 | 9 |
| Fish | | | | | | | | | | | | |
| Moderator | **Subcategory** | **ALA** | **EPA** | **DHA** | **Σω3** | **LA** | **AA** | **Σω6** | **PUFA** | **MUFA** | **SFA** | **ω3:ω6** |
| Body size (cm) | >103 | 22 | 25 | 25 | 22 | 21 | 25 | 22 | 22 | 22 | 22 | 22 |
|  | ≤35 | 27 | 39 | 39 | 30 | 27 | 39 | 30 | 39 | 39 | 39 | 32 |
|  | 35-103 | 28 | 41 | 40 | 17 | 39 | 19 | 17 | 35 | 35 | 35 | 37 |
| Climate | cosmopolitan | 18 | 23 | 22 | NA | 23 | 5 | NA | 18 | 18 | 18 | 18 |
|  | polar | 26 | 32 | 32 | 26 | 26 | 32 | 26 | 34 | 34 | 34 | 28 |
|  | temperate | 33 | 44 | 44 | 37 | 38 | 40 | 37 | 38 | 38 | 38 | 39 |
|  | tropical | NA | 6 | 6 | 6 | NA | 6 | 6 | 6 | 6 | 6 | 6 |
| Duration | >2920.8 | 17 | 32 | 32 | 32 | 32 | 32 | 32 | 29 | 29 | 29 | 32 |
|  | ≤240 | 24 | 30 | 30 | 26 | 16 | 26 | 26 | 30 | 30 | 30 | 26 |
|  | 240-2920.8 | 36 | 43 | 42 | 11 | 39 | 25 | 11 | 37 | 37 | 37 | 33 |
| Feeding type | macrofauna | 50 | 65 | 65 | 42 | 55 | 43 | 42 | 60 | 60 | 60 | 62 |
|  | plankton | NA | 6 | 6 | 7 | 6 | 6 | 7 | 7 | 7 | 7 | 7 |
|  | variable | 27 | 34 | 33 | 20 | 26 | 34 | 20 | 29 | 29 | 29 | 22 |
| Life stage | adult | 42 | 54 | 54 | 45 | 48 | 54 | 45 | 54 | 54 | 54 | 47 |
|  | juvenile | 35 | 45 | 44 | 20 | 39 | 23 | 20 | 38 | 38 | 38 | 40 |
|  | larvae | NA | 6 | 6 | 4 | NA | 6 | 4 | 4 | 4 | 4 | 4 |
| Tissue type | liver | 17 | 24 | 24 | 14 | 15 | 16 | 14 | 27 | 27 | 27 | 20 |
|  | muscle | 26 | 33 | 33 | 25 | 32 | 25 | 25 | 33 | 33 | 33 | 33 |
|  | other | 21 | 26 | 26 | 19 | 24 | 26 | 19 | 19 | 19 | 19 | 21 |
|  | whole | 13 | 22 | 21 | 11 | 16 | 16 | 11 | 17 | 17 | 17 | 17 |
| Resilience | High | NA | 2 | 2 | 2 | NA | 2 | 2 | 2 | 2 | 2 | 2 |
|  | Low | 6 | 18 | 18 | 21 | 14 | 18 | 21 | 21 | 21 | 21 | 21 |
|  | Medium | 71 | 85 | 84 | 46 | 73 | 63 | 46 | 73 | 73 | 73 | 68 |
| Control temp | NA | 77 | 105 | 104 | 69 | 87 | 83 | 69 | 96 | 96 | 96 | 91 |
| Longevity | NA | 73 | 89 | 88 | 53 | 81 | 67 | 53 | 80 | 80 | 80 | 75 |
| Mean temp | NA | 77 | 105 | 104 | 68 | 87 | 83 | 68 | 95 | 95 | 95 | 90 |
| Temp range | NA | 77 | 105 | 104 | 68 | 87 | 83 | 68 | 95 | 95 | 95 | 90 |
| Trophic level | NA | 77 | 105 | 104 | 69 | 87 | 83 | 69 | 96 | 96 | 96 | 91 |
| Vulnerability | NA | 77 | 105 | 104 | 69 | 87 | 83 | 69 | 96 | 96 | 96 | 91 |
| Vulnerability climate | NA | 48 | 64 | 63 | 37 | 58 | 42 | 37 | 55 | 55 | 55 | 57 |
| Primary producers | | | | | | | | | | | | |
| Concentrations | | | | | | | | | | | | |
| Moderator | **Subcategory** | **ALA** | **EPA** | **DHA** | **Σω3** | **LA** | **AA** | **Σω6** | **PUFA** | **MUFA** | **SFA** | **Total FA** |
| Climate | cosmopolitan | 32 | 29 | 49 | 12 | 33 | 14 | 12 | 42 | 44 | 44 | 44 |
|  | polar | 19 | 17 | 12 | NA | 15 | 5 | NA | 13 | 13 | 13 | 13 |
|  | temperate | 98 | 197 | 97 | 67 | 28 | 80 | 25 | 177 | 177 | 177 | 195 |
|  | tropical | 1 | 4 | 13 | NA | 2 | 2 | NA | 4 | 4 | 4 | 16 |
| Duration | >336 | 47 | 69 | 29 | 32 | 21 | 14 | NA | 63 | 63 | 63 | 69 |
|  | ≤228 | 41 | 89 | 67 | 26 | 23 | 39 | 16 | 76 | 61 | 61 | 81 |
|  | 228-336 | 47 | 80 | 40 | 24 | 18 | 68 | 24 | 82 | 84 | 84 | 92 |
| Functional group | diatom | 33 | 106 | 70 | 7 | 21 | 13 | 1 | 94 | 79 | 79 | 86 |
|  | macro | 45 | 63 | 10 | 68 | 7 | 31 | 36 | 76 | 78 | 78 | 78 |
|  | other microalgae | 103 | 139 | 115 | 7 | 58 | 88 | 3 | 123 | 123 | 123 | 150 |
| Life stage | stage1 | NA | 3 | 1 | NA | NA | 3 | NA | 3 | 3 | 3 | 3 |
|  | stage2 | 69 | 131 | 106 | 13 | 38 | 62 | 3 | 115 | 115 | 115 | 140 |
|  | stage3 | 106 | 130 | 71 | 33 | 34 | 35 | 1 | 132 | 117 | 117 | 117 |
| Habitat | benthic | 45 | 92 | 28 | 68 | 29 | 36 | 36 | 97 | 99 | 99 | 101 |
|  | other | 7 | 9 | 19 | NA | 3 | NA | NA | 7 | 7 | 7 | 21 |
|  | pelagic | 125 | 204 | 144 | 10 | 54 | 92 | 4 | 189 | 174 | 174 | 188 |
| Control temp | NA | 181 | 308 | 195 | 82 | 86 | 132 | 40 | 293 | 280 | 280 | 314 |
| Invertebrates | | | | | | | | | | | | |
| Moderator | **Subcategory** | **ALA** | **EPA** | **DHA** | **Σω3** | **LA** | **AA** | **Σω6** | **PUFA** | **MUFA** | **SFA** | **Total FA** |
| Body size (mm) | >200 | 2 | 2 | 2 | NA | 2 | 2 | NA | 2 | 2 | 2 | 2 |
|  | 0.2-2 | 7 | 17 | 16 | 8 | 7 | 12 | 8 | 14 | 16 | 19 | 17 |
|  | 20-200 | 9 | 13 | 13 | 9 | 13 | 13 | 9 | 13 | 13 | 13 | 13 |
|  | 2-20 | 12 | 13 | 13 | NA | 12 | 12 | NA | 12 | 12 | 12 | 12 |
| Climate | temperate | 12 | 19 | 18 | 11 | 12 | 13 | 11 | 19 | 19 | 22 | 20 |
|  | tropical | 18 | 22 | 22 | 6 | 22 | 22 | 6 | 22 | 24 | 24 | 24 |
| Duration | >720 | 2 | 6 | 6 | 2 | 2 | 6 | 2 | 2 | 2 | 2 | 2 |
|  | ≤168 | 18 | 22 | 22 | 4 | 22 | 22 | 4 | 24 | 24 | 24 | 24 |
|  | 168-720 | 10 | 16 | 16 | 9 | 10 | 10 | 9 | 15 | 15 | 18 | 15 |
| Life stage | adult | 16 | 26 | 25 | 15 | 20 | 21 | 15 | 27 | 29 | 32 | 30 |
|  | juvenile | 2 | 3 | 3 | 2 | 2 | 2 | 2 | 2 | 2 | 2 | 2 |
|  | larvae | 12 | 12 | 12 | NA | 12 | 12 | NA | 12 | 12 | 12 | 12 |
| Diet group | carnivore | 2 | 2 | 2 | 2 | 2 | 2 | 2 | 2 | 2 | 2 | 2 |
|  | herbivore | 14 | 20 | 19 | 5 | 14 | 19 | 5 | 14 | 16 | 19 | 17 |
|  | omnivore | 14 | 23 | 23 | 10 | 18 | 18 | 10 | 23 | 23 | 23 | 23 |
| Habitat | benthic | 13 | 22 | 21 | 9 | 17 | 22 | 9 | 17 | 17 | 17 | 18 |
|  | pelagic | 17 | 23 | 23 | 8 | 17 | 17 | 8 | 24 | 26 | 29 | 26 |
| Control temp | NA | 30 | 45 | 44 | 17 | 34 | 39 | 17 | 41 | 43 | 46 | 44 |
| Mean temp | NA | NA | NA | NA | NA | NA | NA | NA | NA | NA | NA | NA |
| Temp range | NA | NA | NA | NA | NA | NA | NA | NA | NA | NA | NA | NA |
| Fish | | | | | | | | | | | | |
| Moderator | **Subcategory** | **ALA** | **EPA** | **DHA** | **Σω3** | **LA** | **AA** | **Σω6** | **PUFA** | **MUFA** | **SFA** | **Total FA** |
| Body size (cm) | >103 | 11 | 11 | 11 | 9 | 9 | 11 | 9 | 4 | 9 | 9 | 4 |
|  | ≤35 | 6 | 12 | 12 | 6 | 8 | 12 | 6 | 12 | 12 | 12 | 12 |
|  | 35-103 | 2 | 12 | 12 | 2 | 8 | 12 | 2 | 2 | 2 | 2 | 4 |
| Climate | cosmopolitan | NA | 2 | 2 | NA | NA | 2 | NA | NA | NA | NA | 2 |
|  | polar | 6 | 6 | 6 | NA | 6 | 6 | NA | 6 | 6 | 6 | 6 |
|  | temperate | 13 | 15 | 15 | 11 | 11 | 15 | 11 | 6 | 11 | 11 | 6 |
|  | tropical | NA | 12 | 12 | 6 | 8 | 12 | 6 | 6 | 6 | 6 | 6 |
| Duration | ≤240 | 9 | 15 | 15 | 10 | 10 | 15 | 10 | 14 | 14 | 14 | 14 |
|  | 240-2920.8 | 4 | 8 | 8 | 2 | 4 | 8 | 2 | 4 | 4 | 4 | 6 |
|  | >2920.8 | 6 | 12 | 12 | 5 | 11 | 12 | 5 | NA | 5 | 5 | NA |
| Feeding type | macrofauna | 13 | 25 | 25 | 15 | 17 | 25 | 15 | 10 | 15 | 15 | 10 |
|  | variable | 6 | 10 | 10 | 2 | 8 | 10 | 2 | 8 | 8 | 8 | 10 |
| Life stage | adult | 11 | 11 | 11 | 5 | 11 | 11 | 5 | 6 | 11 | 11 | 6 |
|  | juvenile | 8 | 18 | 18 | 8 | 14 | 18 | 8 | 8 | 8 | 8 | 8 |
|  | larvae | NA | 6 | 6 | 4 | NA | 6 | 4 | 4 | 4 | 4 | 6 |
| Tissue type | liver | 8 | 9 | 9 | 3 | 9 | 9 | 3 | 9 | 9 | 9 | 9 |
|  | muscle | NA | 7 | 7 | 1 | 7 | 7 | 1 | 1 | 1 | 1 | 1 |
|  | other | 4 | 6 | 6 | 4 | 4 | 6 | 4 | 4 | 4 | 4 | 4 |
|  | whole | 7 | 13 | 13 | 9 | 5 | 13 | 9 | 4 | 9 | 9 | 6 |
| Resilience | High | NA | 2 | 2 | 2 | 2 | 2 | 2 | 2 | 2 | 2 | 2 |
|  | Low | 6 | 6 | 6 | 4 | 4 | 6 | 4 | 4 | 4 | 4 | 4 |
|  | Medium | 13 | 27 | 27 | 11 | 19 | 27 | 11 | 12 | 17 | 17 | 14 |
| Control temp | NA | 19 | 35 | 35 | 17 | 25 | 35 | 17 | 18 | 23 | 23 | 20 |
| Longevity | NA | 15 | 19 | 19 | 7 | 13 | 19 | 7 | 8 | 13 | 13 | 10 |
| Mean temp | NA | 19 | 35 | 35 | 17 | 25 | 35 | 17 | 18 | 23 | 23 | 20 |
| Temp range | NA | 19 | 35 | 35 | 17 | 25 | 35 | 17 | 18 | 23 | 23 | 20 |
| Trophic level | NA | 19 | 35 | 35 | 17 | 25 | 35 | 17 | 18 | 23 | 23 | 20 |
| Vulnerability | NA | 19 | 35 | 35 | 17 | 25 | 35 | 17 | 18 | 23 | 23 | 20 |
| Vulnerability climate | NA | 13 | 23 | 23 | 11 | 17 | 23 | 11 | 6 | 11 | 11 | 8 |

**Table S7.** Heterogeneity and model summary statistics for warming effects on FA proportions and concentrations in primary producers, invertebrates, and fish. QE is the Q statistic for residual heterogeneity (Q statistic), and QM is the Q statistic for the test of moderators (warming level), with significance indicated by asterisks (* p < 0.05; ** p <0.01; *** p <0.001). I^2^ values indicate the percentage of total variation in effect sizes (overall or across papers, taxa, experiments, and effect sizes) that is attributable to true heterogeneity rather than sampling error. Marginal (R^2^_m_) and conditional (R^2^_m_) R^2^ values are shown.

| Proportion | | | | | | | | | |
| --- | --- | --- | --- | --- | --- | --- | --- | --- | --- |
| Primary producers | | | | | | | | | |
| FA | **QE** | **QM** | **I^2^ _total_** | **I^2^ _papers_** | **I^2^ _taxa_** | **I^2^ _experiment_** | **I^2^ _effect size_** | **R^2^ _m_** | **R^2^ _c_** |
| ALA | 12680 **** | 1.76 | 99.47 | 0.00 | 18.91 | 41.90 | 38.66 | 0.01 | 0.62 |
| EPA | 32145 **** | 7.66 *** | 99.20 | 6.19 | 28.21 | 24.20 | 40.60 | 0.02 | 0.60 |
| DHA | 8536 **** | 12.38 **** | 97.93 | 38.60 | 19.06 | 7.61 | 32.67 | 0.05 | 0.68 |
| Σω3 | 825 **** | 20.03 **** | 96.21 | 62.79 | 15.55 | 9.57 | 8.30 | 0.15 | 0.93 |
| LA | 15952 **** | 4.22 * | 99.25 | 12.41 | 62.09 | 0.00 | 24.75 | 0.01 | 0.75 |
| AA | 9275 **** | 0.24 | 99.36 | 0.00 | 37.85 | 0.00 | 61.51 | 0.00 | 0.38 |
| Σω6 | 1082 **** | 0.82 | 99.78 | 98.46 | 1.05 | 0.00 | 0.27 | 0.00 | 1.00 |
| PUFA | 25489 **** | 9.88 **** | 99.59 | 9.12 | 49.86 | 2.33 | 38.28 | 0.03 | 0.63 |
| MUFA | 17511 **** | 2.22 | 99.12 | 7.01 | 58.83 | 11.35 | 21.93 | 0.00 | 0.78 |
| SFA | 42142 **** | 9.96 **** | 99.15 | 32.98 | 16.94 | 13.38 | 35.85 | 0.03 | 0.65 |
| Σω3: Σω6 | 1791 | 10.24 **** | 98.32 | 75.33 | 9.61 | 2.72 | 10.67 | 0.04 | 0.90 |
| Invertebrates | | | | | | | | | |
| FA | **QE** | **QM** | **I^2^ _total_** | **I^2^ _papers_** | **I^2^ _taxa_** | **I^2^ _experiment_** | **I^2^ _effect size_** | **R^2^ _m_** | **R^2^ _c_** |
| ALA | 687 **** | 0.47 | 95.21 | 0.00 | 52.69 | 0.00 | 42.52 | 0.01 | 0.56 |
| EPA | 1881 **** | 5.93 ** | 98.52 | 0.00 | 14.00 | 0.00 | 84.52 | 0.12 | 0.24 |
| DHA | 1947 **** | 2.52 | 98.45 | 12.72 | 0.00 | 62.00 | 23.73 | 0.03 | 0.77 |
| Σω3 | 557 **** | 0.58 | 96.46 | 28.37 | 0.00 | 0.00 | 68.09 | 0.03 | 0.32 |
| LA | 2218 **** | 17.93 **** | 99.18 | 0.00 | 43.39 | 0.00 | 55.78 | 0.32 | 0.62 |
| AA | 537 **** | 4.71 * | 98.13 | 65.43 | 0.00 | 24.33 | 8.37 | 0.04 | 0.92 |
| Σω6 | 214 **** | 37.43 **** | 96.02 | 46.73 | 0.00 | 0.00 | 49.29 | 0.69 | 0.84 |
| PUFA | 570 **** | 3.82 * | 99.00 | 10.90 | 0.00 | 67.50 | 20.60 | 0.04 | 0.80 |
| MUFA | 449 **** | 2.46 | 97.02 | 0.00 | 21.84 | 58.12 | 17.06 | 0.02 | 0.83 |
| SFA | 364 **** | 6.55 ** | 92.20 | 0.00 | 35.75 | 19.17 | 37.28 | 0.13 | 0.65 |
| Σω3: Σω6 | 236 **** | 2.71 | 93.33 | 0.00 | 0.00 | 0.00 | 93.33 | 0.16 | 0.16 |
| Fish | | | | | | | | | |
| FA | **QE** | **QM** | **I^2^ _total_** | **I^2^ _papers_** | **I^2^ _taxa_** | **I^2^ _experiment_** | **I^2^ _effect size_** | **R^2^ _m_** | **R^2^ _c_** |
| ALA | 730 **** | 0.03 | 95.33 | 27.18 | 27.18 | 0.00 | 40.98 | 0.00 | 0.57 |
| EPA | 3407 **** | 2.03 | 99.59 | 34.71 | 0.00 | 41.04 | 23.84 | 0.03 | 0.77 |
| DHA | 2385 **** | 0.13 | 98.29 | 20.90 | 0.00 | 52.78 | 24.60 | 0.00 | 0.75 |
| Σω3 | 2365 **** | 0.65 | 99.33 | 0.00 | 0.00 | 76.75 | 22.58 | 0.01 | 0.77 |
| LA | 890 **** | 0.21 | 98.84 | 59.85 | 23.22 | 0.00 | 15.77 | 0.00 | 0.84 |
| AA | 769 **** | 0.66 | 92.48 | 41.84 | 0.00 | 34.19 | 16.45 | 0.02 | 0.83 |
| Σω6 | 627 **** | 0.64 | 91.63 | 52.58 | 0.00 | 1.93 | 37.12 | 0.03 | 0.61 |
| PUFA | 3817 **** | 0.48 | 98.98 | 0.00 | 3.83 | 62.57 | 32.58 | 0.01 | 0.67 |
| MUFA | 5486 **** | 0.25 | 99.21 | 0.00 | 0.00 | 47.45 | 51.76 | 0.01 | 0.48 |
| SFA | 692 **** | 0.11 | 93.80 | 43.97 | 0.00 | 3.96 | 45.87 | 0.00 | 0.51 |
| Σω3: Σω6 | 614 **** | 10.23 *** | 86.22 | 6.58 | 0.00 | 31.13 | 48.51 | 0.17 | 0.53 |
| Concentration | | | | | | | | | |
| Primary producers | | | | | | | | | |
| FA | **QE** | **QM** | **I^2^ _total_** | **I^2^ _papers_** | **I^2^ _taxa_** | **I^2^ _experiment_** | **I^2^ _effect size_** | **R^2^ _m_** | **R^2^ _c_** |
| ALA | 12697 **** | 4.67 * | 99.63 | 0.00 | 29.84 | 34.14 | 35.65 | 0.03 | 0.65 |
| EPA | 52304 **** | 9.19 *** | 99.11 | 43.40 | 0.00 | 21.30 | 34.41 | 0.03 | 0.66 |
| DHA | 6523 **** | 5.13 ** | 98.93 | 43.82 | 10.88 | 11.78 | 32.45 | 0.03 | 0.68 |
| Σω3 | 4923 **** | 6.51 ** | 98.65 | 46.34 | 0.00 | 22.52 | 29.79 | 0.08 | 0.72 |
| LA | 9164 **** | 0.21 | 99.06 | 57.29 | 29.56 | 7.07 | 5.14 | 0.00 | 0.95 |
| AA | 6072 **** | 1.04 | 99.41 | 80.86 | 6.38 | 2.73 | 9.45 | 0.00 | 0.91 |
| Σω6 | 2740 **** | 0.18 | 99.01 | 51.43 | 31.30 | 8.38 | 7.89 | 0.00 | 0.92 |
| PUFA | 35186 **** | 14.84 **** | 99.70 | 0.00 | 48.54 | 21.19 | 29.97 | 0.05 | 0.71 |
| MUFA | 38187 **** | 0.09 | 98.92 | 2.88 | 34.76 | 15.79 | 45.49 | 0.00 | 0.54 |
| SFA | 24442 **** | 0.21 | 99.66 | 5.09 | 33.50 | 14.61 | 46.47 | 0.00 | 0.53 |
| Total FA | 16922 **** | 3.77 * | 99.21 | 14.62 | 38.48 | 12.55 | 33.57 | 0.02 | 0.67 |
| Invertebrates | | | | | | | | | |
| FA | **QE** | **QM** | **I^2^ _total_** | **I^2^ _papers_** | **I^2^ _taxa_** | **I^2^ _experiment_** | **I^2^ _effect size_** | **R^2^ _m_** | **R^2^ _c_** |
| ALA | 138 **** | 0.94 | 97.47 | 41.64 | 41.64 | 4.63 | 9.56 | 0.01 | 0.90 |
| EPA | 814 **** | 1.09 | 97.50 | 56.59 | 0.00 | 0.00 | 40.91 | 0.04 | 0.60 |
| DHA | 1269 **** | 1.63 | 97.97 | 40.12 | 0.00 | 27.22 | 30.63 | 0.05 | 0.70 |
| Σω3 | 100 **** | 0.21 | 92.36 | 7.04 | 7.04 | 17.34 | 60.95 | 0.01 | 0.35 |
| LA | 228 **** | 1.84 | 93.57 | 50.28 | 15.54 | 20.50 | 7.25 | 0.04 | 0.93 |
| AA | 408 **** | 0.21 | 97.08 | 43.12 | 0.00 | 24.47 | 29.49 | 0.01 | 0.70 |
| Σω6 | 57 **** | 0.59 | 80.15 | 0.00 | 0.00 | 0.00 | 80.15 | 0.05 | 0.05 |
| PUFA | 565 **** | 0.02 | 95.75 | 44.64 | 0.00 | 0.00 | 51.11 | 0.00 | 0.47 |
| MUFA | 744 **** | 0.11 | 98.49 | 17.49 | 0.00 | 75.24 | 5.77 | 0.00 | 0.94 |
| SFA | 938 **** | 0.21 | 96.89 | 42.49 | 0.00 | 25.79 | 28.61 | 0.01 | 0.71 |
| Total FA | 1118 **** | 0.5 | 97.52 | 59.03 | 0.00 | 0.00 | 38.48 | 0.01 | 0.61 |
| Fish | | | | | | | | | |
| FA | **QE** | **QM** | **I^2^ _total_** | **I^2^ _papers_** | **I^2^ _taxa_** | **I^2^ _experiment_** | **I^2^ _effect size_** | **R^2^ _m_** | **R^2^ _c_** |
| ALA | 73 **** | 0.12 | 86.24 | 0.00 | 42.01 | 27.60 | 16.63 | 0.01 | 0.81 |
| EPA | 326 **** | 0.23 | 91.88 | 0.00 | 0.00 | 87.77 | 4.12 | 0.00 | 0.96 |
| DHA | 259 **** | 5.15 * | 87.80 | 0.00 | 63.62 | 0.00 | 24.18 | 0.22 | 0.79 |
| Σω3 | 30 ** | 0 | 73.20 | 30.92 | 30.92 | 11.37 | 0.00 | 0.00 | 1.00 |
| LA | 132 **** | 0.57 | 88.18 | 29.26 | 29.26 | 0.18 | 29.49 | 0.02 | 0.67 |
| AA | 103 **** | 8.1 ** | 68.62 | 0.00 | 0.00 | 42.41 | 26.22 | 0.41 | 0.78 |
| Σω6 | 37 *** | 0.01 | 85.46 | 39.73 | 39.73 | 6.00 | 0.00 | 0.00 | 1.00 |
| PUFA | 115 **** | 0.02 | 89.80 | 27.71 | 27.71 | 0.00 | 34.38 | 0.00 | 0.62 |
| MUFA | 306 **** | 0.19 | 91.89 | 8.57 | 8.57 | 0.00 | 74.76 | 0.01 | 0.20 |
| SFA | 73 **** | 0.04 | 78.58 | 10.67 | 10.67 | 0.00 | 57.25 | 0.00 | 0.27 |
| Total FA | 342 **** | 3.1 | 98.80 | 0.00 | 0.00 | 19.31 | 79.49 | 0.25 | 0.40 |

**Table S8**. Heterogeneity and model summary statistics for acidification effects on FA proportions and concentrations in primary producers, invertebrates, and fish. QE is the Q statistic for residual heterogeneity (Q statistic), and QM is the Q statistic for the test of moderators (acidification level), with significance indicated by asterisks (* p < 0.05; ** p <0.01; *** p <0.001). I^2^ values indicate the percentage of total variation in effect sizes (overall or across papers, taxa, experiments, and effect sizes) that is attributable to true heterogeneity rather than sampling error. Marginal (R^2^_m_) and conditional (R^2^_m_) R^2^ values are shown.

| Proportion | | | | | | | | | |
| --- | --- | --- | --- | --- | --- | --- | --- | --- | --- |
| Primary producers | | | | | | | | | |
| FA | **QE** | **QM** | **I^2^ _total_** | **I^2^ _papers_** | **I^2^ _taxa_** | **I^2^ _experiment_** | **I^2^ _effect size_** | **R^2^ _m_** | **R^2^ _c_** |
| ALA | 5860 **** | 0.61 | 99.59 | 0.00 | 80.88 | 17.52 | 1.19 | 0.03 | 0.99 |
| EPA | 409 **** | 0.19 | 95.54 | 79.52 | 0.67 | 0.00 | 15.35 | 0.01 | 0.84 |
| DHA | 212 **** | 0.55 | 93.32 | 61.44 | 0.00 | 0.00 | 31.88 | 0.02 | 0.67 |
| Σω3 | 3 | 1.35 | 0.00 | 0.00 | 0.00 | 0.00 | 0.00 | 1.00 | 1.00 |
| LA | 5575 **** | 1.42 | 99.80 | 36.15 | 60.55 | 0.00 | 3.11 | 0.01 | 0.97 |
| AA | 67 **** | 2.29 | 92.56 | 43.22 | 43.22 | 1.55 | 4.57 | 0.05 | 0.95 |
| Σω6 | 1 | 0.23 | 0.00 | 0.00 | 0.00 | 0.00 | 0.00 | 1.00 | 1.00 |
| PUFA | 5703 **** | 0.25 | 99.30 | 0.00 | 97.03 | 0.64 | 1.63 | 0.00 | 0.98 |
| MUFA | 12487 **** | 1.55 | 99.60 | 0.00 | 98.37 | 0.00 | 1.24 | 0.01 | 0.99 |
| SFA | 4684 **** | 3.49 * | 98.57 | 6.82 | 72.83 | 18.92 | 0.00 | 0.03 | 1.00 |
| Σω3: Σω6 | 6278 **** | 0.96 | 99.01 | 0.00 | 99.01 | 0.00 | 0.00 | 0.01 | 1.00 |
| Invertebrates | | | | | | | | | |
| FA | **QE** | **QM** | **I^2^ _total_** | **I^2^ _papers_** | **I^2^ _taxa_** | **I^2^ _experiment_** | **I^2^ _effect size_** | **R^2^ _m_** | **R^2^ _c_** |
| ALA | 229 **** | 3.73 * | 86.92 | 30.00 | 0.00 | 0.00 | 56.92 | 0.14 | 0.44 |
| EPA | 173 **** | 2.04 | 90.31 | 0.00 | 0.00 | 70.91 | 19.40 | 0.06 | 0.80 |
| DHA | 852 **** | 0.41 | 99.13 | 0.00 | 0.00 | 98.95 | 0.18 | 0.00 | 1.00 |
| Σω3 | 14 | 0.13 | 35.20 | 10.57 | 10.57 | 0.00 | 14.06 | 0.09 | 0.64 |
| LA | 239 **** | 0.83 | 96.91 | 46.94 | 7.25 | 0.00 | 42.72 | 0.03 | 0.57 |
| AA | 100 **** | 2.15 | 82.78 | 0.00 | 0.00 | 0.00 | 82.78 | 0.17 | 0.17 |
| Σω6 | 23 * | 2.24 | 41.06 | 0.00 | 0.00 | 41.06 | 0.00 | 0.65 | 1.00 |
| PUFA | 836 **** | 0.78 | 99.71 | 0.00 | 0.00 | 81.40 | 18.31 | 0.01 | 0.82 |
| MUFA | 538 **** | 1.11 | 98.76 | 0.00 | 0.00 | 0.00 | 98.76 | 0.06 | 0.06 |
| SFA | 205 **** | 3.74 * | 97.28 | 38.71 | 0.00 | 29.31 | 29.25 | 0.12 | 0.74 |
| Σω3: Σω6 | 90 **** | 0.23 | 95.11 | 0.00 | 0.00 | 95.11 | 0.00 | 0.01 | 1.00 |
| Fish | | | | | | | | | |
| FA | **QE** | **QM** | **I^2^ _total_** | **I^2^ _papers_** | **I^2^ _taxa_** | **I^2^ _experiment_** | **I^2^ _effect size_** | **R^2^ _m_** | **R^2^ _c_** |
| EPA | 53 **** | 0.47 | 95.86 | 21.90 | 21.90 | 6.44 | 45.61 | 0.11 | 0.58 |
| DHA | 55 **** | 0.38 | 97.87 | 0.00 | 0.00 | 14.12 | 83.75 | 0.07 | 0.20 |
| LA | 60 **** | 0.15 | 94.29 | 0.00 | 0.00 | 0.00 | 94.29 | 0.03 | 0.03 |
| AA | 655 **** | 0.27 | 99.06 | 0.00 | 0.00 | 0.00 | 99.06 | 0.04 | 0.04 |
| PUFA | 18 * | 0.01 | 96.11 | 45.18 | 45.18 | 0.00 | 5.75 | 0.00 | 0.94 |
| MUFA | 50 **** | 0.05 | 96.41 | 32.11 | 32.11 | 0.00 | 32.19 | 0.01 | 0.67 |
| SFA | 17 * | 1.24 | 60.44 | 0.00 | 0.00 | 0.00 | 60.44 | 0.24 | 0.24 |
| Concentration | | | | | | | | | |
| Primary producers | | | | | | | | | |
| FA | **QE** | **QM** | **I^2^ _total_** | **I^2^ _papers_** | **I^2^ _taxa_** | **I^2^ _experiment_** | **I^2^ _effect size_** | **R^2^ _m_** | **R^2^ _c_** |
| ALA | 14597 **** | 2.51 | 99.77 | 0.00 | 93.33 | 0.00 | 6.44 | 0.09 | 0.94 |
| EPA | 109 **** | 4.08 * | 86.18 | 0.00 | 56.81 | 0.00 | 29.37 | 0.45 | 0.81 |
| DHA | 106 **** | 5.9 ** | 96.47 | 79.55 | 0.00 | 16.92 | 0.00 | 0.09 | 1.00 |
| Σω3 | 12 | 0.03 | 43.41 | 0.00 | 21.91 | 21.51 | 0.00 | 0.00 | 1.00 |
| LA | 75 **** | 2.7 | 87.29 | 0.00 | 0.00 | 73.21 | 14.08 | 0.35 | 0.90 |
| AA | 6 | 0 | 29.34 | 9.78 | 9.78 | 0.00 | 9.78 | 0.00 | 0.67 |
| Σω6 | 7 | 0.01 | 25.20 | 25.20 | 0.00 | 0.00 | 0.00 | 0.01 | 1.00 |
| PUFA | 18749 **** | 17.59 **** | 99.89 | 13.70 | 81.63 | 4.55 | 0.00 | 0.06 | 1.00 |
| MUFA | 1663 | 20.98 **** | 97.87 | 51.34 | 0.00 | 44.97 | 1.57 | 0.52 | 0.99 |
| SFA | 2718 **** | 1.26 | 99.36 | 28.16 | 0.00 | 71.20 | 0.00 | 0.12 | 1.00 |
| Total FA | 5634 **** | 7.15 ** | 99.70 | 33.28 | 53.35 | 13.07 | 0.00 | 0.16 | 1.00 |
| Invertebrates | | | | | | | | | |
| FA | **QE** | **QM** | **I^2^ _total_** | **I^2^ _papers_** | **I^2^ _taxa_** | **I^2^ _experiment_** | **I^2^ _effect size_** | **R^2^ _m_** | **R^2^ _c_** |
| ALA | 9 | 0.16 | 40.25 | 17.82 | 17.82 | 4.60 | 0.00 | 0.04 | 1.00 |
| EPA | 28 * | 5.39 * | 57.31 | 0.00 | 0.00 | 57.31 | 0.00 | 0.53 | 1.00 |
| DHA | 430 **** | 1.61 | 97.99 | 0.00 | 0.00 | 97.99 | 0.00 | 0.01 | 1.00 |
| Σω3 | 4 | 1.42 | 59.94 | 0.00 | 0.00 | 59.94 | 0.00 | 0.25 | 1.00 |
| LA | 35 **** | 0.42 | 87.75 | 36.44 | 36.44 | 0.00 | 14.87 | 0.03 | 0.84 |
| AA | 8 | 0.06 | 0.00 | 0.00 | 0.00 | 0.00 | 0.00 | 1.00 | 1.00 |
| Σω6 | 2 | 0.01 | 34.23 | 0.00 | 0.00 | 34.23 | 0.00 | 0.00 | 1.00 |
| PUFA | 172 **** | 5.54 * | 91.53 | 0.00 | 0.00 | 78.30 | 13.23 | 0.21 | 0.89 |
| MUFA | 244 **** | 0.05 | 98.46 | 35.42 | 35.42 | 10.15 | 17.47 | 0.00 | 0.82 |
| SFA | 299 **** | 0.4 | 96.60 | 26.98 | 26.98 | 30.83 | 11.81 | 0.01 | 0.88 |
| Total FA | 531 **** | 0.91 | 97.81 | 39.15 | 39.15 | 17.13 | 2.38 | 0.01 | 0.98 |
| Fish | | | | | | | | | |
| FA | **QE** | **QM** | **I^2^ _total_** | **I^2^ _papers_** | **I^2^ _taxa_** | **I^2^ _experiment_** | **I^2^ _effect size_** | **R^2^ _m_** | **R^2^ _c_** |
| EPA | 226 **** | 0.49 | 97.83 | 0.00 | 0.00 | 0.00 | 97.83 | 0.08 | 0.08 |
| DHA | 226 **** | 2.58 | 98.56 | 5.36 | 5.36 | 0.00 | 87.83 | 0.33 | 0.41 |
| AA | 712 **** | 0.37 | 99.78 | 34.75 | 34.75 | 0.00 | 30.29 | 0.10 | 0.73 |
| PUFA | 371 **** | 0.45 | 99.15 | 10.84 | 10.84 | 0.00 | 77.48 | 0.11 | 0.30 |
| MUFA | 240 **** | 0 | 98.92 | 10.73 | 10.73 | 0.00 | 77.46 | 0.00 | 0.22 |
| SFA | 112 **** | 0.06 | 97.55 | 9.70 | 9.70 | 13.69 | 64.46 | 0.02 | 0.35 |
| Total FA | 4 | 0.32 | 23.55 | 9.39 | 9.39 | 4.77 | 0.00 | 0.25 | 1.00 |

**Table S9 (see “Table_S9_SuppInfo.xlsx”).** Heterogeneity and model summary statistics for the effects of warming on FA proportions and concentrations in primary producers, invertebrates, and fish, with additional biological or experimental covariates included. QE is the Q statistic for residual heterogeneity (Q statistic), and QM is the Q statistic for the test of moderators (acidification level), with significance indicated by asterisks (* p < 0.05; ** p <0.01; *** p <0.001). I^2^ values indicate the percentage of total variation in effect sizes (overall or across papers, taxa, experiments, and effect sizes) that is attributable to true heterogeneity rather than sampling error. Marginal (R^2^_m_) and conditional (R^2^_m_) R^2^ values are shown. Rows in bold indicate significant covariate effects (p<0.05; ANOVA for categorical, estimate/slope β for continuous).
